# Role of oxidative stress and inflammation-related signaling pathways in doxorubicin-induced cardiomyopathy

**DOI:** 10.1186/s12964-023-01077-5

**Published:** 2023-03-14

**Authors:** Saixian Shi, Ye Chen, Zhijian Luo, Guojun Nie, Yan Dai

**Affiliations:** 1 Department of Pharmacy, Affiliated Hospital of Southwest Medical University, No. 25 Taiping Street, Luzhou, 646000 Sichuan Province China; 2 School of Pharmacy, Southwest Medical University, Luzhou, 646000 Sichuan Province China; 3 Department of Ultrasound, The Affiliated Hospital of Southwest Medical University, Luzhou, 646000 Sichuan Province China; 4 The First Outpatient Department of People’s Liberation Army Western Theater General Hospital, Chengdu, 610000 Sichuan Province China

**Keywords:** Doxorubicin, Cardiomyopathy, Oxidative stress, Inflammation, Signaling pathway, Nrf2, NF-κB

## Abstract

Doxorubicin (DOX) is a powerful and commonly used chemotherapeutic drug, used alone or in combination in a variety of cancers, while it has been found to cause serious cardiac side effects in clinical application. More and more researchers are trying to explore the molecular mechanisms of DOX-induced cardiomyopathy (DIC), in which oxidative stress and inflammation are considered to play a significant role. This review summarizes signaling pathways related to oxidative stress and inflammation in DIC and compounds that exert cardioprotective effects by acting on relevant signaling pathways, including the role of Nrf2/Keap1/ARE, Sirt1/p66Shc, Sirt1/PPAR/PGC-1α signaling pathways and NOS, NOX, Fe^2+^ signaling in oxidative stress, as well as the role of NLRP3/caspase-1/GSDMD, HMGB1/TLR4/MAPKs/NF-κB, mTOR/TFEB/NF-κB pathways in DOX-induced inflammation. Hence, we attempt to explain the mechanisms of DIC in terms of oxidative stress and inflammation, and to provide a theoretical basis or new idea for further drug research on reducing DIC.

**Video Abstract**

**Supplementary Information:**

The online version contains supplementary material available at 10.1186/s12964-023-01077-5.

## Introduction

Doxorubicin (DOX) is a classical chemotherapy agent derived from anthracycline, whose anticancer effect has been confirmed in decades of clinical application, extensively used alone or in combination for the treatment of breast cancer, lymphoma, acute leukemia, ovarian cancer and other cancers [1]. DOX plays a significant anticancer role by acting on topoisomerase IIα and inhibiting synthesis of DNA and RNA. However, obvious adverse cardiac reaction is often in the wake of the anticarcinogenic effect of DOX, clinically manifested as elevated troponin, arrhythmia, myocarditis and cardiomyopathy, which seriously restricted the clinical application. Cardiomyopathy can be classified as dilated, hypertrophic and restrictive according to the clinical phenotype [2]. DOX-induced cardiomyopathy (DIC) is a non-ischemic cardiomyopathy often presenting as dilated [3], clinically presented with left ventricular expansion accompanied by systolic dysfunction that cannot be explained by pressure or volume overload or coronary artery disease [4]. There is a strong correlation between cumulative doses of DOX and incidence and severity of DIC, in addition, age and previous cardiovascular disease were found to increase the incidence of DIC. Severe cardiomyopathy cause progressive heart failure and irreversible cardiac dysfunction, even death, severely affecting the quality of survival of cancer survivors [5].

With the development of the detection technology, echocardiography, cardiac computed tomography, cardiac magnetic resonance imaging, nuclear and molecular cardiology and monitoring strategies for cardiac biomarkers have been progressively applied in clinical practice to facilitate early diagnosis and treatment of patients [6]. Biomarkers used to detect DOX-induced cardiomyopathy include cardiac troponin I (cTnI), hypersensitive troponin I, creatine kinase isoenzyme (CK-MB), type B brain natridium peptide (BNP) and NT-pro BNP [7].

The current clinical treatment strategies for DOX-induced cardiomyopathy are mainly to limit the cumulative dose of DOX, the use of liposomal doxorubicin and the application of cardioprotective drugs, including Dexrazoxane, angiotensin converting enzyme inhibitor (ACEI), angiotensin receptor blocker (ARB), mineralocorticoid receptor antagonist (MRA), β-blockers (BB) and statins [6, 8]. Dexrazoxane (DEX) is the only drug currently approved to prevent DOX-induced cardiomyopathy by inhibiting topoisomerase IIβ and DOX-Fe^2+^ complex [9]. Moreover, DEX can also alleviate DOX-induced apoptosis by upregulating the expression of miR-17-5p and inhibiting p38 mitogen-activated protein kinases (p38 MAPK)/nuclear factor-kappaB (NF-κB) pathway [10, 11]. However, one study showed that more than 59% of patients who received DEX pretreatment had elevated high-sensitivity troponin T levels, suggesting that DEX pretreatment could not completely improve DIC [12]. Therefore, it is crucial to further investigate the molecular mechanisms of DIC and find more effective targeted therapeutic strategies.

**Fig. 1:**
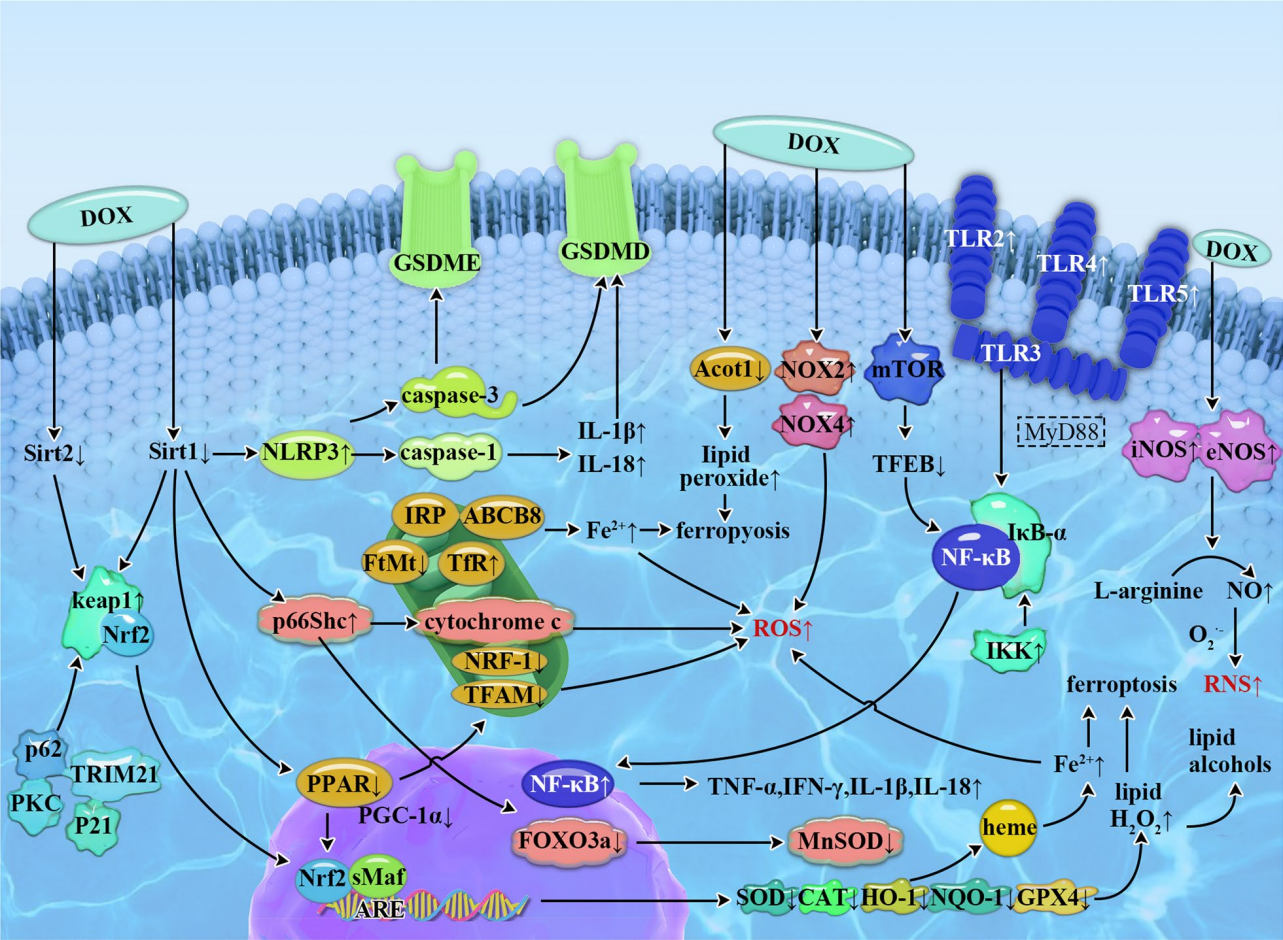
Schematic representation of oxidative stress and inflammation-related signaling pathways in doxorubicin-induced cardiomyopathy. DOX induces overgeneration of ROS and RNS and leads to oxidative stress by activating Nrf2/Keap1/ARE, SIRT1/p66Shc, Sirt1/PPAR/PGC-1α pathway as well as interfering with NOS, NOX and Fe^2+^ signaling. DOX increases the secretion and release of inflammatory cytokines by acting on NLRP3/caspase-1/GSDMD, HMGB1/TLR4/MAPKs/NF-κB, mTOR/TFEB/NF-κB pathway, and further cause cell and tissue damage. DOX: doxorubicin, ROS: reactive oxygen species, RNS: reactive nitrogen species, Sirt1: Silent information regulator 1, Nrf2: Nuclear factor E2-related factor 2, Keap1: kelch-like ECH associated protein 1, sMaf: small Maf proteins, ARE: antioxidant response element, NQO-1: NAD(P)H quinone oxidoreductase-1, SOD: superoxide dismutase, GPX: Glutathione peroxidase 4, HO-1: heme oxygenase-1, P66Shc: The 66-kDa Src homology 2 domain-containing protein, NOX: NAD(P)H oxidase, iNOS: inducible nitric oxide synthas, eNOS: endothelial nitric oxide synthase, PPAR: Peroxisome proliferator-activated receptors, PGC-1α: PPAR coactivator 1α, NRF-1: nuclear respiratory factor 1, TFAM: mitochondrial transcription factor A, Acot1: acyl-coenzyme A thioesterase 1, IRP: iron regulins protein, FtMt: Mitochondrial ferritin, TfR: transferrin receptor, ABCB8: ATP-binding cassette transporter protein B8, NLRP3: nucleotide-binding domain-like receptor protein 3, GSDMD: gasdermin D, GSDME: gasdermin E, MyD88: myeloid differentiation factor 88, IRF3: interferon regulator 3, NF-κB: nuclear factor-κB, TLR: Toll-like receptors, TNF-α: tumor necrosis factor-α, IL: interleukin, IKK: IκB kinase, mTOR: Mechanistic target of rapamycin, TFEB: transcription factor EB

In recent years, the molecular mechanism of DIC has been extensively studied, including oxidative stress, inflammatory response, mitochondrial dysfunction, autophagy, apoptosis, myocardial fibrosis, Ca^2+^ overload, endoplasmic reticulum stress and so on [13–16]. It has been found that DIC was resulted by a variety of mechanisms, involving multiple signal pathways. This review article summarizes signaling pathways related to oxidative stress and inflammatory (Fig. 1), and lists some drugs that play a cardioprotective role in DIC by acting on signaling pathways.

## Signaling pathways related to oxidative stress

Oxidative stress refers to the stress state caused by the unbalance between the weakening of body antioxidant defense system and the excessive generation of reactive oxygen species (ROS) and reactive nitrogen species (RNS), which is one of the fundamental causes for DIC. Cardiolipin is a type of membrane phospholipid that resides in the inner lobe of the mitochondrial membrane in cardiomyocytes, which has high affinity with DOX. The high affinity between DOX and cardiolipin makes DOX easy to accumulate in mitochondria and lead to mitochondrial dysfunction mediated by oxidative stress [17]. The combination of DOX and cardiolipin can interrupt electron transfer chain (ETC) via inhibiting the activity of complexes I, II and IV [18]. The quinone structure in DOX can be reduced to semiquinone intermediate, catalyzed by several oxidoreductases, such as cytochrome P450 reductase, xanthine oxidase, nicotinamide adenine dinucleotide phosphate (NAD(P)H) oxidase (NOX) through a single-electron reduction mechanism. The hemiquinone intermediates rapidly reduces oxygen to uperoxide(O_2_^·−^), which can be transformed into hydrogen peroxide (H_2_O_2_) via the catalysis of manganese superoxide dismutase (MnSOD or SOD). In contrast, H_2_O_2_ is less toxic. However, H_2_O_2_ can be further converted into hydroxyl radical (^·^OH) with stronger activity and toxicity in the presence of Fe^2+^ [19]. The accumulation of DOX in mitochondria can bring about excessive generation of ROS (including O_2_^·−^, H_2_O_2_ and ^·^OH), leading to mitochondrial protein oxidation, lipid peroxidation, as well as DNA damage, further leading to sarcolemmal and mitochondrial sarcoplasmic reticular change, which eventually causes myocardial contraction impairment [20–22]. ROS generation and mitochondrial damage promote mutually, and elevated ROS levels can directly lead to ETC inactivation and mitochondrial dysfunction, further increasing the generation of ROS [23].

RNS refers to a series of radical and nitro compounds with high oxidative activity derived from the interaction of NO and compounds including reactive oxygen species, such as peroxynitrite anion (ONOO^−^) et al. The response between NO and O_2_^·−^ most likely leads to NO depletion, which impairs the endothelium-dependent vasodilatory function [24]. Some important biomolecules such as proteins, lipids and DNA react with ONOO^−^ through direct or radical-mediated mechanisms, leading to changes in enzymatic activity and signaling pathways. ONOO^−^ has a high affinity for tyrosine residues in proteins and can form nitrolated proteins by nitrating tyrosine groups, resulting in loss or enhancement of enzymatic activity [25]. The reaction of ONOO^−^ with DNA may lead to the production of multiple oxidation products of the purine and pyrimidine bases, such as 8-nitroguanine, a biomarker of oxidative DNA damage [26]. Moreover, it was found that DOX increased the generation of ONOO^−^ in myocytes, which subsequently caused the activation of c-Jun N-terminal kinase (JNK), thereby increasing the expression of High mobility group box 1(HMGB1) in cardiomyocytes and involved in DOX-induced cardiomyocyte apoptosis [27]. Overproduction of peroxynitrite overcomes the endogenous antioxidant mechanism, which ultimately disrupts cellular homeostasis and leads to cell death.

Besides, the effects of DOX include a significant reduction in the level of endogenous antioxidant enzymes, such as SOD, NAD(P)H quinone oxidoreductase-1 (NQO-1), heme oxygenase-1 (HO-1), glutathione peroxidase (GPX), catalase (CAT) and so on, which significantly weaken the body's antioxidant defense system and cause the imbalance of redox [28].

### Nrf2/Keap1/ARE signaling pathway

**Fig. 2:**
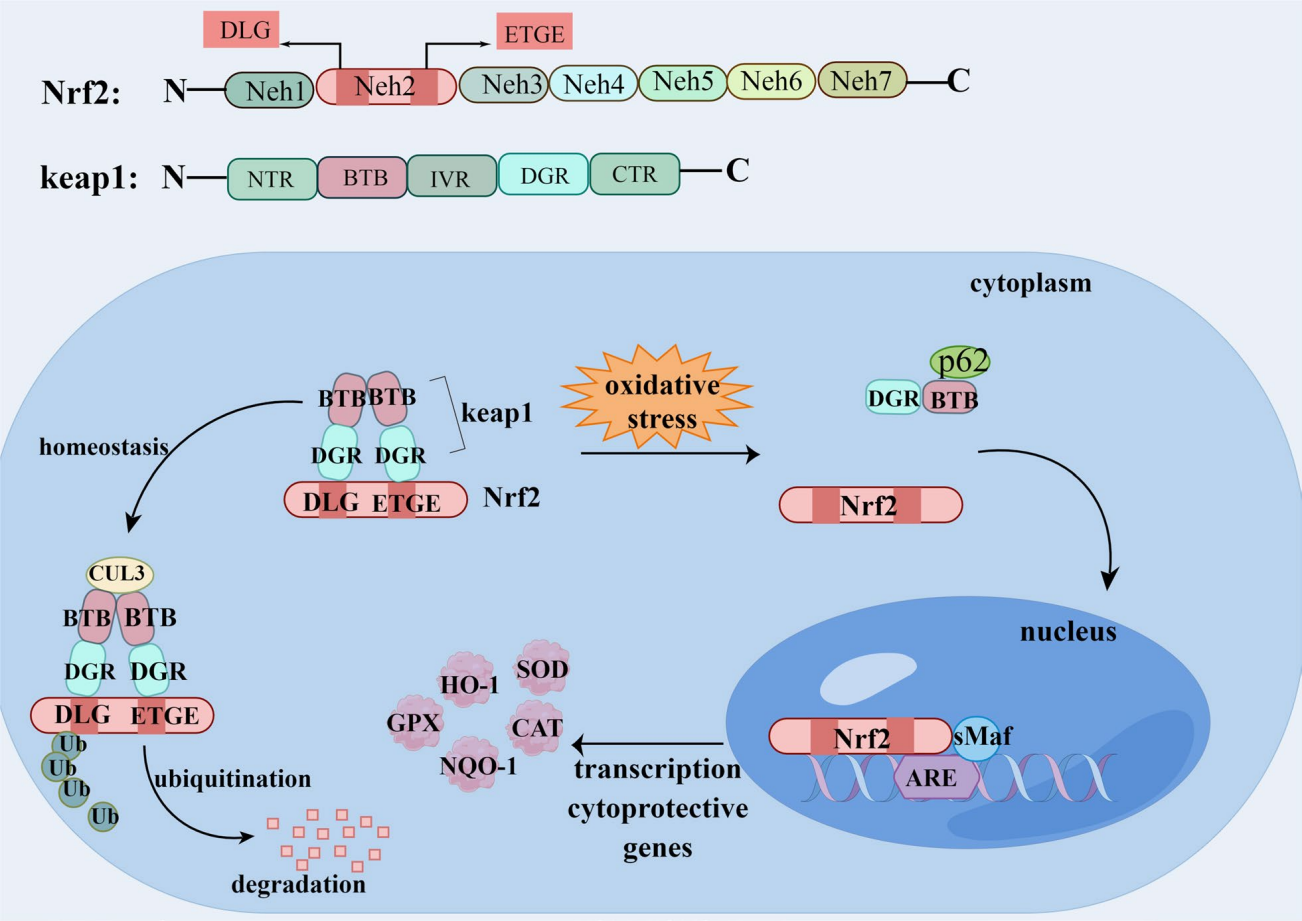
Schematic diagram of the mechanism of Nrf2/Keap1/ARE signaling pathway. Nrf2 has seven homodomains, Neh1–7. Keap1 has five domains containing NTR, BTB, IVR, DGR, and CTR. Nrf2 binds to the DGR domain of keap1 homodimer via the DLG and ETGE fragments, and Cul3 binds to the BTB domain of Keap1. Under basal conditions, Nrf2 is ubiquitinated and degraded by the Keap1-Cul3 complex, without generating biological activity. Upon stimulated, Nrf2 dissociates from the Keap1-Nrf2 complex, ectopically into the nucleus and binds to sMaf, and Nrf2-sMaf binds to ARE to promote the expression of antioxidant genes, such as NQO-1, SOD, GPX, HO-1. Nrf2: Nuclear factor E2-related factor 2, Keap1: kelch-like ECH associated protein 1, sMaf: small Maf proteins, ARE: antioxidant response element, NQO-1: NAD(P)H quinone oxidoreductase-1, SOD: superoxide dismutase, GPX: Glutathione peroxidase 4, HO-1: heme oxygenase-1. (By Figdraw.)

Nuclear factor E2-related factor 2 (Nrf2) is a critical regulator of various physiological and pathological processes, playing a key role in regulating cellular redox state [29]. Nrf2 is not biologically active and does not activate downstream genes in normal physiological state. Nrf2 has seven homodomains, Neh1–7, of which the Neh2 domain contains both DLG and ETGE fragments, as required for interaction with kelch-like ECH associated protein 1 (keap1) [30]. Nrf2 widely exists in cells as a compound formed by combining with keap1. Keap1 has five domains containing NTR, BTB, IVR, DGR, and CTR. Among them, the BTB domain allows cullin3 and keap1 binding, and the DGR domain is essential for the interaction of Keap1 with other proteins (e.g.Nrf2 and p62) [30]. Keap1 promotes the ubiquitination and degradation of Nrf2 in the cytoplasm under the action of E3 ubiquitin ligase containing cullin3. Lack of Keap1 causes elevated Nrf2 activity and further raised the expression of downstream antioxidant genes [31]. When cells are stimulated, Nrf2 is stripped from the Nrf2-Keap1 complex and transfers to the nucleus, binds to the small Maf proteins (sMaf). Nrf2 interacts with the antioxidant response element (ARE) of cytoprotective genes with the help of sMaf to activate the downstream expression of antioxidase, including SOD, CAT, HO-1, NAD(P)H oxidase and so on [30, 32] (Fig. 2). It was found that the expressions of Nrf2 and HO-1 were slightly upregulated and the expression of Keap1 gene was inhibited in the early stage of DOX treatment, but this weak upregulation was not enough to offset the oxidative stress induced by doxorubicin [33, 34]. Over all, DOX treatment can increase Keap1 level, inhibit expression of Nrf2, HO-1, NAD(P)H oxidase and aggravate oxidative stress [35, 36]. Nrf2 deficiency can even aggravate the damage caused by DOX [37].

Silent information regulator 1(Sirt1) deacetylates Nrf2 in order to activate it [38]. Knockout of Sirt1 downregulated the expression of Nrf2 and HO-1 [39]. Besides, Sirt2 is also involved in regulating the expression of Nrf2. Study has found that miR-140-5p was obviously increased after DOX treatment, which could aggravate oxidative stress by targeting Sirt2/Nrf2 [40]. Dioscin directly reduces the expression of miR-140-5p and activates Nrf2/Sirt2 signaling pathways, subsequently increasing the expression of antioxidant enzyme and reducing ROS generation [41]. The p62/Sqstm1 protein can release Nrf2 by binding to Keap1, increasing Nrf2 activity and enhancing the expression of antioxidant genes [42]. Researchers have found that expression of tripartite motif containing-21 (TRIM21) is upregulated after DOX treatment, adversely affecting the function of Nrf2. TRIM21 is an E3 ubiquitin ligase that interacts with p62 to disturb the separation of Nrf2 from Nrf2-keap1 and inhibit the activity of Nrf2, further decreasing the expression of downstream antioxidant genes. Inhibition or knockdown of TRIM21 could enhance Nrf2 expression and attenuated DIC [43]. In addition, protein kinase C (PKC) [44], P13K/Akt [45], p21 protein [46] can also activate Nrf2 to phosphorylate by interacting with Keap1 [47].

Researchers have proved that alpha-Linolenic acid, Sulforaphane, and Asiatic Acid can play a protective role in DIC by activating Keap1/Nrf2/ARE pathway [48–50]. Resolvin D1, Orosomucoid 1, Punicalagin, Fisetin and others improved cellular redox defense and alleviated DIC by activating the Nrf2/HO-1 pathway [34, 39, 51–61]. Baicalein, β-lapachone, Indole-3-carbinol, p-Coumaric acid alleviates DIC by activating Nrf2/ARE pathway and enhancing antioxidant enzymes expression (including HO-1, SOD, CAT, GST, NQO1 and GPX) in the myocardium [49, 54, 62–69]. At present, a variety of compounds that can act on Keap1/Nrf2/ARE signaling pathway have been found (Additional file 1: Table S1). Nrf2 is expected to become a therapeutic target against DIC [70].

### Sirt1/p66Shc signaling pathway

P66Shc (The 66-kDa Src homology 2 domain-containing protein) is a member of the adapter protein family involved in several biological processes such as ROS synthesis, proliferation and apoptosis, containing a highly conserved N-terminal phosphotyrosine binding domain (PTB),central proline-enriched region 1 (CH1), C-terminal Src identity region 2 (SH2), cytochrome c binding domain and a unique CH2 domain [71]. The presence of the ser36 amino acid residues in the CH2 domain largely determines the cell sensitivity to ROS [71]. The p66Shc is in an inactive state in normal physiological state. During oxidative stress, on the one hand, p66Shc can be transferred to the nucleus, transported to mitochondria as well as related membranes and combined with mitochondrial cytochrome c, resulting in the oxidation of cytochrome c, further promoting the generation of ROS [72]. On the other hand, p66Shc is activated by the phosphorylation of ser36 amino acid residues and activates AKT (a serine/threonine protein kinase) to inactivate FOXO3a transcription factors, thus reducing the level of MnSOD and reducing the cellular detoxification of ROS [73, 74].

Studies have shown that Sirt1 is related to regulating the expression of p66Shc, and the level of p66Shc is reduced in rats with knockout of Sirt1 gene, while the level of p66Shc can be restored by transfection of Sirt1 gene [75]. Sirt1 is a nicotinamide adenine dinucleotide (NAD +) dependent enzyme which can catalyze the deacetylation of lysine residues of histone and participate in the regulation of multiple vital movement such as proliferation, growth and activation [76, 77]. Expression of p66Shc requires the involvement of acetylated histones, while Sirt1 can reduce the amount of acetylated histones by catalyzing histone deacetylation, thereby reducing p66Shc expression and decreasing its activity [78].

Researchers demonstrated that p66Shc has a hand in the oxidative stress process [72], and downregulation or knockdown of p66Shc alleviated oxidative stress and ROS generation in rat cells [74]. In contrast, overexpression of p66Shc exacerbates oxidative stress. After DOX treatment, increased p66Shc content and decreased Sirt1 expression were observed in both in vitro and in vivo experiments. Wu et al. showed that berberine can downregulate the expression of p66shc by activating Sirt1,enhance the body's antioxidant defense (including SOD, CAT, GPX),and promote lipid H_2_O_2_ metabolize into malondialdehyde to alleviate DIC [79]. Zhu et al. showed that the level of miR-34a-5p increased after DOX treatment, and miR-34a-5p could increase the heart injury induced by DOX by targeting Sirt1 and activating Sirt1/p66Shc pathway, while blocking this pathway could achieve the purpose of cardiac protection [80]. Liu et al. found that the expression of intracellular miR-124 decreased after DOX treatment, increasing the expression of miR-124 can alleviate oxidative stress and cardiac injury by inhibiting p66Shc [81] (Additional file 7: Table S7). The Sirt1/p66shc signaling pathway may be a therapeutic target for alleviate DIC.

### NOX singnaling

NOX is the cytochrome subunit of the phagocyte NAD(P)H oxidase, playing a crucial role in the generation of ROS [82]. The NOX family is composed of NOX1-5 and DUOX1,2, of which NOX2 and NOX4 are mostly existed in cardiomyocytes and regulate of cardiomyocyte function. NOX2 and NOX4 can reduce the quinone structure of DOX to hemiquinone intermediate through a single-electron reduction mechanism, which converts O_2_ into O_2_^·−^,H_2_O_2_ and ^·^OH through a series of reactions, resulting in myocardial injury [21]. NOX itself has no catalytic activity and requires binding with subunits to form a stable complex to exert catalysis. Five subunits take part in the activation of NOX2, including p22phox, p67phox, p40phox, p47phox, as well as the GTPase Rac, in which p22phox plays a major role. The activation of NOX4 requires the involvement of p22phox and polymerase Poldip2 [83, 84]. DOX has been shown to activate NOX signaling, promotes NOX2 and NOX4 expression and ROS generation, exacerbate oxidative stress and further activating apoptosis mediated by MAPK [85]. In addition, Dox activates the motility-related protein 1 (Drp1) by enhancing the expression of NOX1 and NOX4, further inducing mitochondrial division, and causing the NLRP3 inflammasome-mediated pyroptosis in cardiomyocytes [86]. It was found that knockdown of NOX2 and NOX4 can prevent excessive generation of ROS and attenuated DIC [87, 88].

**Fig. 3:**
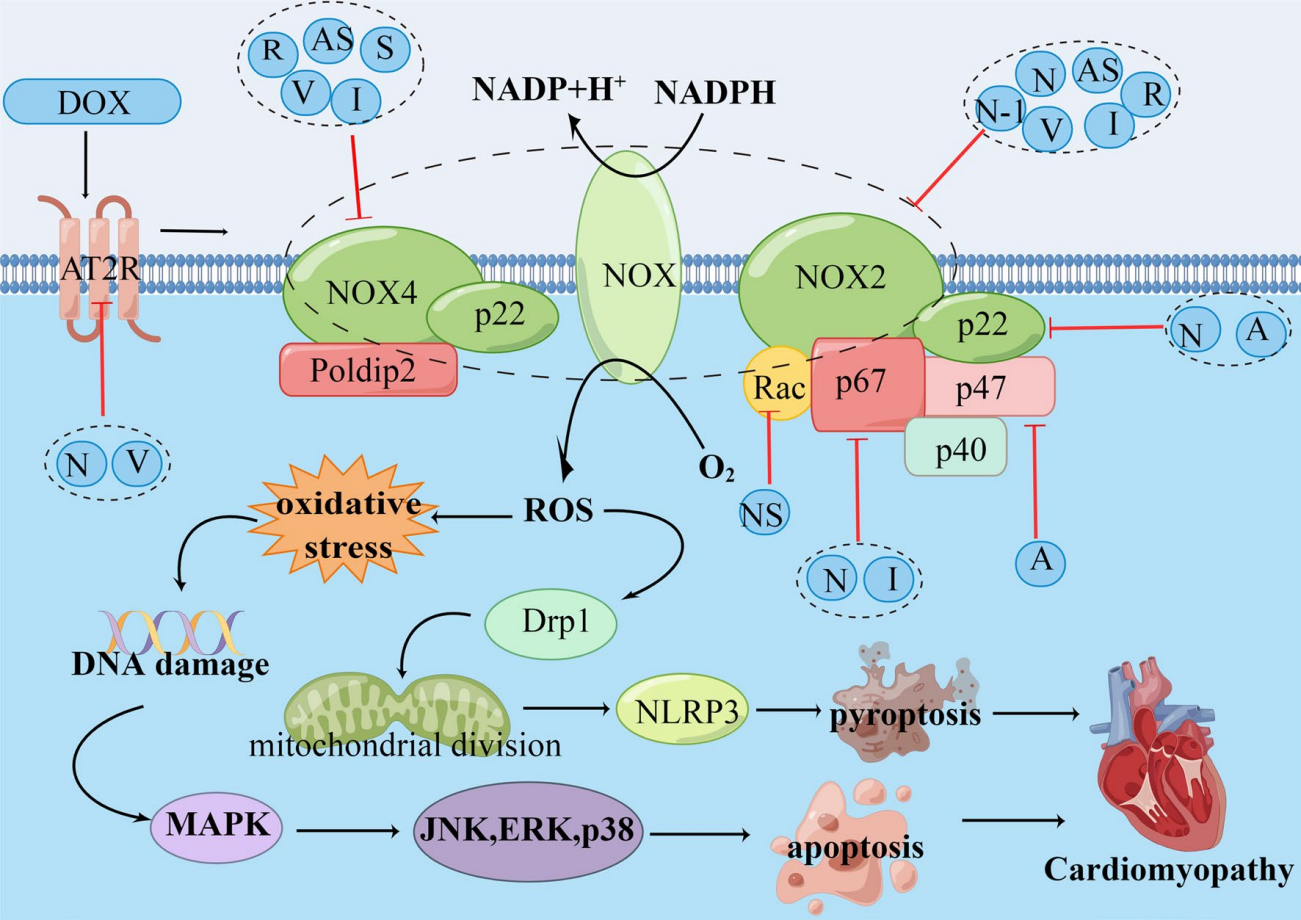
Schematic representation of the role of NAD(P)H oxidase (NOX) in DOX-induced cardiomyopathy. DOX upregulates NOX2 and NOX4 expression by activating the angiotensin receptor, oxidizes NADPH, and reduces O_2_ to produce ROS. On the one hand, ROS causes oxidative stress and DNA damage, further activates MAPK-mediated apoptosi. On the other hand, ROS activates Drp1, inducing mitochondrial division, causing NLRP3-mediated apoptosis and eventually causing myocardial damage. Natural compounds including neferine (N), valsartan (V), necrostain-1 (N-1), setanaxib (S), astragaloside (AS), acacia (A), irisin (I), NSC23766 (NS) and resolvin D1 (R) attenuated DOX-induced cardiomyopathy by downregulation of NOX2 and NOX4. DOX: doxorubicin, Drp1: motility-related protein 1, MAPK: mitogen-activated protein kinases, NLRP3: nucleotide- binding domain-like receptor protein 3, ROS: reactive oxygen species. (By Figdraw.)

Many compounds have been found to reduce DIC by inhibiting activity of NOX [83] (Additional file 2: Table S2). Neferine, astragaloside IV, acacia hydaspica and resolvin D1 can exert a cardioprotective effect by inhibiting NOX [21, 55, 88–91]. As an momentous regulator, Rac are involved in the activation of NOX2. The activation of Rac can trigger the feedback self-activation of NOX2, resulting in oxidation burst and obvious increase of ROS production [92]. Conversely, knockout of Rac can inhibit the activation of NOX and cut down the generation of ROS, and mitigate DIC. Giving specific Rac inhibitor NSC23766 to mice with DIC can also achieve cardiac protection [93]. In addition, p67phox also take part in the activation of NOX2. Experiments by Zhang et al. have proved that irisin can reduce the activation of NOX, inhibit the activity of NOX and reduce oxidative stress by inhibiting the expression of p67phox [94]. Angiotensin II (Ang II) can activate and regulate the expression of NOX. Valsartan can reduce the DIC by inhibiting AngII receptor and downregulating the expression of NOX2 and NOX4 [85] (Fig. 3).

### NOS singnaling

**Fig. 4:**
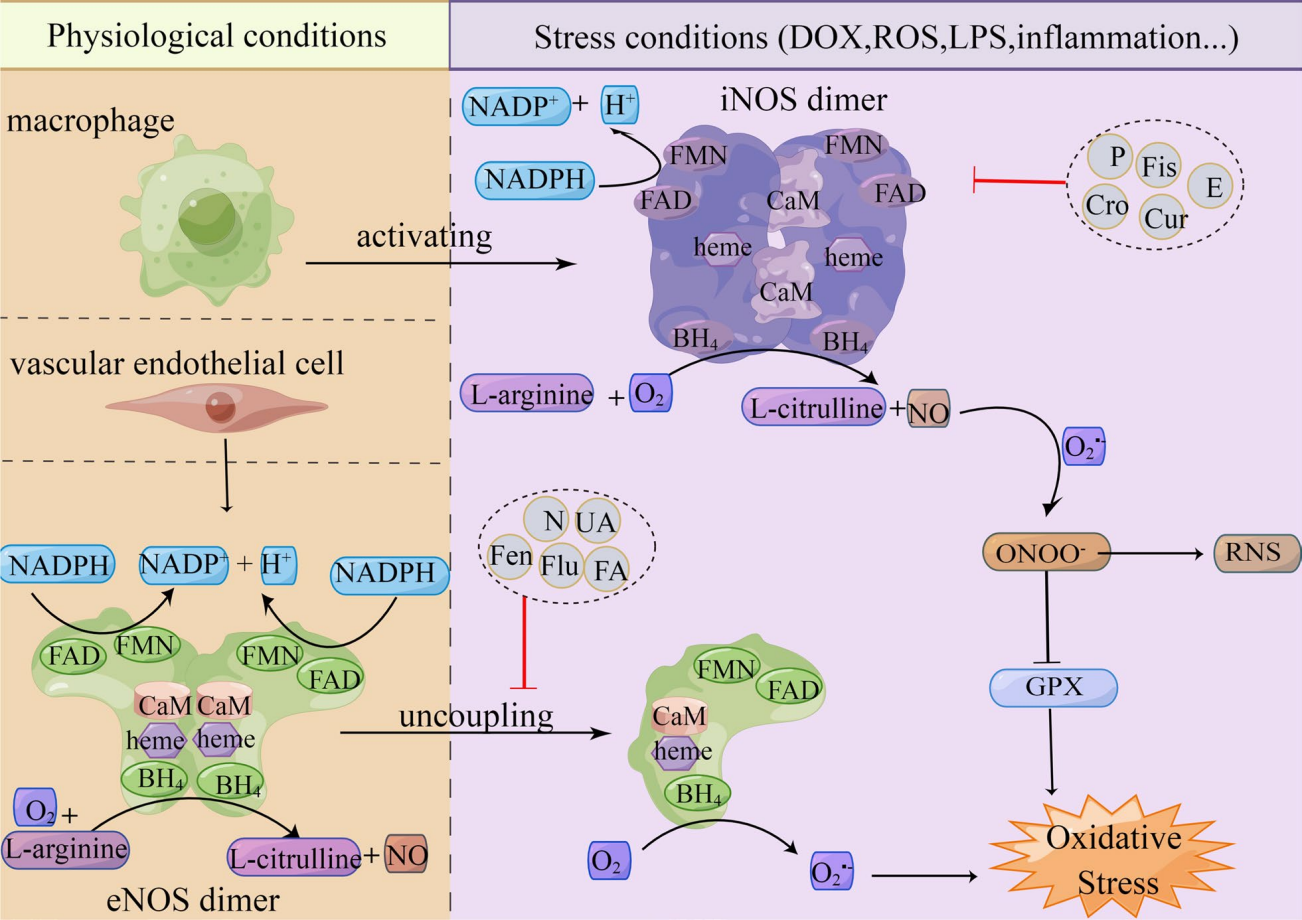
Schematic representation of the role of iNOS and eNOS in DOX-induced cardiomyopathy. iNOS is mainly distributed in macrophages and is not expressed under physiological conditions. The iNOS is activated under stress, converts L-arginine to L-citrulline and generates a large amount of NO with the help of the co-factors FAD, FMN, BH4, heme complex-ferriprotoporphyrin IX. NO can react with O_2_^·−^ to generate ONOO^−^, which inhibits GPX and exacerbates oxidative stress. The eNOS is mainly distributed in endothelial cells and formed in its physiological state can reduce O_2_ to NO. In the stress state, eNOS is uncoupled to reduce O_2_ to O_2_^·−^, aggravating the oxidative stress. Drugs including phenylalanine-butyramide(P), fisetin(Fis), curcumin(Cur), crocin(Cro), eicosapentaenoic acid(E) can attenuate oxidative stress by downregulating the activation of iNOS. Drugs including nebivolol(N), fenofibrate(Fen), ursolic acid(UA), folic acid(FA), fluvastatin(Flu) attenuate oxidative stress by downregulating the activation of eNOS. iNOS: inducible nitric oxide synthase, eNOS: endothelial nitric oxide synthase, BH4: tetrahydrobiopterin, FAD: flavin adenine dinucleotide, FMN: flavin mononucleotide, CaM: calmodulin. (By Figdraw.)

Except outside the oxidative stress mediated by ROS, RNS-mediated nitrosative stress also participates in the process of DIC (Fig. 4). RNS is a kind of free radical and nitro compounds with high oxidation activity, generated by the interaction between ROS and nitric oxide(NO). Thus, NO levels largely determine the levels of intracellular RNS and are important in DIC. NO is one of the products of L-arginine catalyzed by nitric oxide synthase (NOS). NOS consists of three isoforms, including neuronal nitric oxide synthase (nNOS), inducible nitric oxide synthase(iNOS) and endothelial nitric oxide synthase (eNOS), in which iNOS and eNOS take part in the catalytic synthesis of NO in the myocardium. NO levels can be directly regulated by NOS, and NO levels are also indirectly affected by endothelin-1(ET-1) activity and ROS levelsN [95]. No acts as a messenger molecule with small molecular weight that can dilate blood vessels. The normal NO concentration and its bioavailability are important for the maintenance of cardiovascular and neural tissue function [96].

The process of NO production catalyzed by iNOS requires the participation of NADPH and four cofactors, including tetrahydrobiopterin (BH4), flavin adenine dinucleotide (FAD), flavin mononucleotide (FMN) and heme complex-ferriprotoporphyrin IX [97]. The iNOS expression is affected by NF-κB and IFN-γ. DOX treatment can upregulate the expression of iNOS and the synthesis of NO. The excess of NO and O_2_^·−^ produce ONOO^−^. Excessive accumulation of free radicals with highly oxidative activity such as ONOO^−^ can affect the expression of GPX and promote the nuclear translocation of NF-κB to induce oxidative stress as well as lipid peroxidation [97–100]. what’s more, iNOS can also mediate ER stress, activate Toll-like receptor-2 (TLR2) to promote inflammation, and further induce apoptosis [101]. The HER2 inhibitor lapatinib exacerbated DIC by increasing the expression of iNOS and the generation of NO [102]. Studies have found a variety of compounds that can exert cardioprotective effects by reducing the expression of iNOS, such as eicosapentaenoic acid, curcumin, crocin, vitamin E, fisetin, liposomal resveratrol, carvedilol, nebivolol and so on [103–108] (Additional file 3: Table S3).

The process of NO production can also be catalyzed by eNOS, whose cofactors required for catalysis are the same as the iNOS. The activation of eNOS is associated with AKT, activation of adenosine monophosphate-activated protein kinase (AMPK) and soluble guanylate cyclase (sGC) [109]. In the physiological state, the eNOS is in a relatively stable dimer-coupled state to generate the NO. During oxidative stress, eNOS uncouples into unstable eNOS monomers that no longer generate NO while generate large amounts of O_2_^·−^, further aggravating oxidative stress. On the one hand, DOX can induce eNOS uncoupling by affecting NOX4, Ang II receptor and AKT, increasing the monomer/dimer eNOS ratio. DOX binds to eNOS monomers to form a hemiquinone, which reacts rapidly with oxygen radicals to increase the production of O_2_^·−^ and reduce NO synthesis as well as bioavailability. On the other hand, DOX inhibits the phosphorylation at eNOS Ser1177, increases the phosphorylation at eNOS Thr495, and decreases the eNOS activity [110]. The results showed that DOX treatment in eNOS knockout rats showed lower ROS levels and weaker cardiotoxicity than the control group. However, myocardial-specific eNOS overexpression can aggravate the cardiac toxicity caused by DOX [111]. Therefore, the inhibition of the eNOS uncoupling and reducing the number of eNOS monomers could be used as a strategy for cardioprotection. Vitamin C can reduce DIC by increasing the level of cofactor BH4 and stabilizing the eNOS and reducing the number of eNOS monomer. Meanwhile, vitamin C also increases the phosphorylation at eNOS Ser1177 to reduce the DOX-induced nitrosative stress [112]. A special amino acid formulation used for promoting cellular respiration modulates phosphorylation at Ser1177 by affecting mTOR complex 1 (mTORC1) and activates eNOS/mTORC1 signaling to prevent DIC [113, 114]. Fenofibrate, urgulic acid and folic acid can increase NO bioavailability by activating eNOS and inhibiting eNOS uncoupling to alleviate DIC [115–117] (Additional file 3: Table S3).

### Sirt1/PPAR/PGC-1α signaling pathway

Peroxisome proliferator-activated receptors (PPAR) is a ligand-inducible nuclear receptor with three isoforms containing PPARα, PPARγ and PPARβ/δ [118]. PPAR is widely expressed in cardiomyocytes and involved in cardiomyocyte energy metabolism, proliferation, differentiation, development as well as the regulation of cell death [119]. It was found that DOX can cause mitochondrial dysfunction by acting on PPAR, activating oxidative stress and inflammation and ultimately inducing apoptosis. On the one hand, DOX can reduce the expression of PPAR by regulating Sirt1 or AMPK and further upregulate NF-κB expression, promote inflammatory factor release and aggravate the inflammatory response [120]. On the other hand, DOX can decrease Nrf2 expression by inhibiting PPAR, reduce the level of antioxidant enzymes and reduce the body’s antioxidant capacity, resulting in oxidative stress [121]. The expression of PPARα, PPARγ and PPARβ/δ in the heart decreased after DOX treatment [122–125]. It was found that Glycyrrhiza Glabra root extract could reduce DIC by restoring Sirt1 and PPARα/γ levels [124]. Piperine, Astragali Radix, Catalpol can exert their cardioprotective effects through the activation of PPAR [126–128].

PPAR coactivator 1α(PGC-1α) is an inducible transcription coactivator of PPAR, which enhances the nuclear transcriptional function of PPAR and plays a significant role in regulating various signaling pathways occurring in mitochondria. The expression of PGC-1α is regulated by AMPK and Sirt1, and Sirt3 [129, 130]. Deacetylation of Sirt1 and Sirt3 can activate PGC-1α to increase the level of its downstream factor Nrf2 and the transcription of the antioxidant gene SOD and HO-1, significantly enhancing the antioxidant defense system of body [131]. DOX treatment decreased the levels of SIRT1 and PGC-1α, reduced the antioxidant capacity and induced oxidative stress. Ferruginol can exert cardioprotective effects by activating PGC-1α expression by acting on Sirt1 [132]. Dichloroacetate attenuates DIC by restoring the abnormal SIRT3 and PGC-1α signaling caused by DOX [133]. Pterostilbene can upregulate PGC-1α activity by activating AMPK and SIRT1 to reduce the oxidative stress caused by DOX [134]. Troxerutin can prevent DOX from downregulating the levels of SIRT1 and PGC-1α and reduce cardiomyocyte damage [121]. PGC-1α can also act as a regulatory factor of the expression of nuclear respiratory factor 1(NRF-1) and mitochondrial transcription factor A (TFAM), thus affecting mitochondrial biosynthesis process [135]. Sonowal et al. have found that fidarestat (an aldose reductase inhibitor) reduced DIC by increasing the levels of PGC-1α and TFAM and enhancing mitochondrial biogenesis [136]. What’s more, PGC-1α can also affect the expression of uncoupling protein 2 (UCP2). UCP2 is an oxidative stress-protective molecule that may reduce oxidative stress by transferring Ca^2+^ from extracellular to matrix and exporting lipid peroxides [137]. Dexmedetomidine can reduce the degradation of PGC-1α and increase the expression of UCP2, significantly reducing the synthesis of mitochondrial ROS [138]. Matrine can also reverse the downregulation of UCP2 caused by DOX through AMPK activation, and reduce the oxidative damage and apoptosis in cardiomyocytes [139] (Additional file 4: Table S4).

The current study has also found that a portion of MicroRNAs (miRNAs), which is abnormally expressed after DOX treatment, aggravate DIC by targeting PPAR or PGC-1α (Additional file 7: Table S7). MiRNAs is a kind of small single stranded noncoding RNA that can lead to the degradation of mRNA and inhibit mRNA translation by acting on the untranslated region of mRNA. In vitro and in vivo experiments found that miR-128-3p and miR-130a were upregulated after DOX treatment, and the inhibition of miR-128-3p and miR-130a produced cardioprotective effects. Moreover, this protective effect can be abolished by PPARγ antagonists, which fully demonstrates that miR-128-3p and miR-130a can target and reduce PPAR expression [125, 140]. MiR-22 is abnormally expressed in DOX-treated cells. Inhibition or knockout of miR-22 can inhibit mitochondrial biosynthesis, reduce ROS production and reduce heart injury. It was found that this protective effect was achieved by activating Sirt1 and upregulating the expression of PGC-1α, TFAM and NRF-1 [135]. In cardiotoxicity models, the expression of miR-23a increased with the cumulative dose of DOX, and miR-23a significantly leads to mitochondrial damage and apoptosis by targeting PGC-1α and the phosphorylation of Dynamin-related protein-1 (Drp1). Inhibition of miR-23a significantly alleviated mitochondrial dysfunction and oxidative stress [141]. Therefore, screening for mRNA and other indicators abnormally expressed after DOX treatment can also be used as a way to study the molecular mechanism of DIC.

### Iron signaling

Fe^2+^ participates in the generation of ROS in mitochondria through Fenton reaction, and then induces the generation and accumulation of oxidized lipids [142]. Iron is one of the fundamental trace elements to maintain normal life activities of the body. Lack or excess of iron will lead to the occurrence of diseases, such as anemia, chronic heart failure and so on [143]. It was found that iron homeostasis imbalance causes overproduction of ROS, induces lipid peroxidation, and eventually leads to cell ferroptosis [144]. Ferroptosis is a regulatory cell death caused by the accumulation of lethal lipid peroxides, which is different from apoptosis and is characterized by intact mitochondrial nuclei but rupture of the outer membrane [145]. Ferrostatin-1(fer-1) removes lipid peroxides by reducing the generated alkoxy groups for anti-iron death effects [146].

On the one hand, DOX can induce ferroptosis by directly interfering with the clearance of lipid peroxides. The formation of Fe^2+^-dependent toxic lipid ROS can be significantly reduced by converting lipid hydrogen peroxide into lipid alcohols, which is less toxic. Glutathione peroxidase 4 (GPX4) plays an important role in this transformation process and inhibition of GPX4 function results in the accumulation of lipid peroxides in Cardiomyocytes [147]. The DOX-Fe^2+^ complex formed by DOX and Fe^2+^ can downregulate the expression of GPX4, reduce the reduction of oxidized phospholipids, and increase the accumulation of oxidized phospholipids in mitochondria [148]. It was found that protein arginine methyltransferase 4 (PRMT4) could regulate the expression of GPX4. PRMT4 overexpression catalyzes the enzymatic methylation of Nrf2, resulting in the restricted nuclear translocation of Nrf2 and reducing the expression of downstream iron death related gene. While knoknockdown of PRMT4 promotes nuclear ectopic of Nrf2 and alleviates cardiac damage [149]. Experiments showed that GPX4 overexpression or iron chelate targeting Fe^2+^ in mitochondria could reduce DOX-induced ferroptosis [144]. Astragaloside IV and Salidroside can play a cardioprotective role by upregulating the expression of GPX4, restoring its antioxidant capacity and reducing the accumulation of oxidized phospholipids [150, 151]. MITOL/MARCH5 is an E3 ubiquitin ligase that inhibits DOX-induced ferroptosis by maintaining the ratio of glutathione/ glutathione disulfide(GSH/GSSG) in mitochondria and the expression of GPX4. Conversely, knockdown of MITOL/MARCH5 aggravated DIC, and this aggravation of cardiomyopathy could be offset by fer-1 [152] In addition to GPX4, acyl-coenzyme A thioesterase 1 (Acot1) is also involved in lipid metabolism, that inhibit lipid peroxidation. It was found that Acot1 was down-regulated after DOX treatment, and the knockdown of Acot1 sensitized cardiomyocytes to ferritin action and aggravated iron death. In contrast, overexpression of Acot1 attenuated DOX-induced ferroptosis [153] (Additional file 7: Table S7).

On the other hand, DOX can disrupt iron homeostasis through the following three pathways, leading to iron overload and subsequently inducing the generation of lipid peroxides. Firstly, DOX leads to iron overload within the mitochondria by increasing iron synthesis and uptake. DOX and its metabolites can interfere with the expression of iron regulins protein 1 and 2 (IRP1 and IRP2), increasing iron absorption and reducing iron storage [154]. ROS is an important factor in mediating the regulation of IRP activity [155]. In addition, the activation of TLR4 and NOX4 can also interfere with the function of the IRP and mediate ferroptosis [156]. Mitochondrial ferritin (FtMt), an iron-storing protein in mitochondria decreases in quantity and leads to increased intracellular free Fe^2+^ content after DOX treatment [157]. DOX can increase the cellular uptake of Fe^2+^ by upregulating the transferrin receptor (TfR). Secondly, DOX interferes with iron release.DOX causes the activation of Nrf-2 to bring about the upregulation of HO-1, catalyzes heme degradation and induce the release of free Fe^2+^, ultimately leading to the accumulation of oxidized lipids in the mitochondrial membrane [158]. Finally, DOX interferes with the excretion of iron. ATP-binding cassette transporter proteinB8 (ABCB8) is a mitochondrial protein that promotes iron excretion.DOX treatment brings about the accumulation of Fe^2+^ within the mitochondria by downregulating ABCB8 expression and reducing Fe^2+^ expulsion [159]. Study has found that ABCB8 deficiency even led to reduced efflux of DOX, causing DOX accumulation and increased DIC. In contrast, ABCB8 overexpression improved intracellular DOX retention and toxicity [160]. Besides, DOX reduces the extracellular excretion of Fe^2+^ by downregulating the iron export protein ferroportin (FPN) expression.

**Fig. 5:**
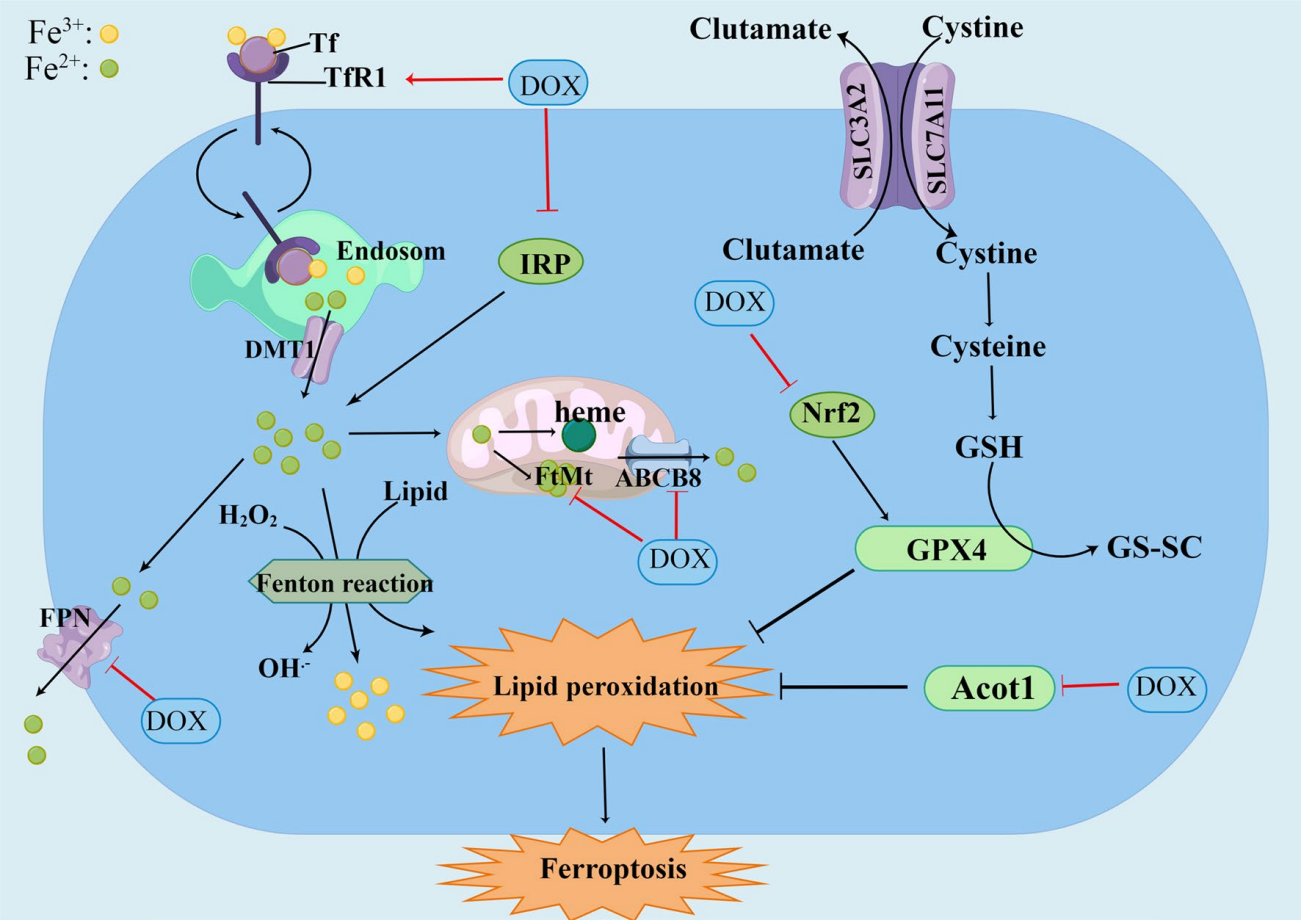
Schematic representation of the mechanism of ferroptosis in DOX-induced cardiomyopathy. Ferroptosis is a type of cell death mediated by lipid peroxidation. Fe^3+^ is imported by the transferrin receptor and converted to Fe^2+^ in endosome, released from endosomes to cytoplasm by the bivalent metal transporter 1. Fe^2+^ is converted to Fe^3+^ by fenton and activates lypoxygenases to induce lipid peroxidation. After Fe^2+^ entering the mitochondria, it can participate in the heme composition, be stored in FtMt, and be excreted in the mitochondria via ABCB8. Free Fe^2+^ can be excreted from the cytoplasm by FPN. Glutathione peroxidase 4 is the main endogenous mechanism to inhibit lipid peroxidation. Glutathione is a cofactor of GPX4. Cells transfer glutamate to extracellular via glutamate inhibit system Xc, at the same time, cystine enters the cell and subsequently transforms to cysteine to produce GSH. DOX can increase uptake of Fe^2+^ by upregulating TfR, and reduce excretion of Fe^2+^ by downregulation of FPN, ABCB8, and FtMt, leading to Fe^2+^ overload-mediated lipid peroxidation. DOX can reduce the clearance of lipid peroxides by downregulation of GPX4 and Acot1, leading to lipid peroxide accumulation-mediated ferroptosis. DOX: doxorubicin, TfR: transferrin receptor, DMT1: bivalent metal transporter 1, FtMt: mitochondrial ferritin, ABCB8: ATP-binding cassette transporter proteinB8, FPN: ferroportin, GPX4: Glutathione peroxidase 4, GSH: Glutathione, IRP: Iron regulins protein. (By Figdraw.)

Fe^2+^ overload in cardiomyocytes can mediate lipid peroxidation and further induce cell ferroptosis, playing a crucial role in DIC [161]. Therefore, inhibition of lipid peroxidation and maintenance of iron homeostasis are important for mitigate DIC (Fig. 5).

## Signaling pathways related to inflammation

**Fig. 6:**
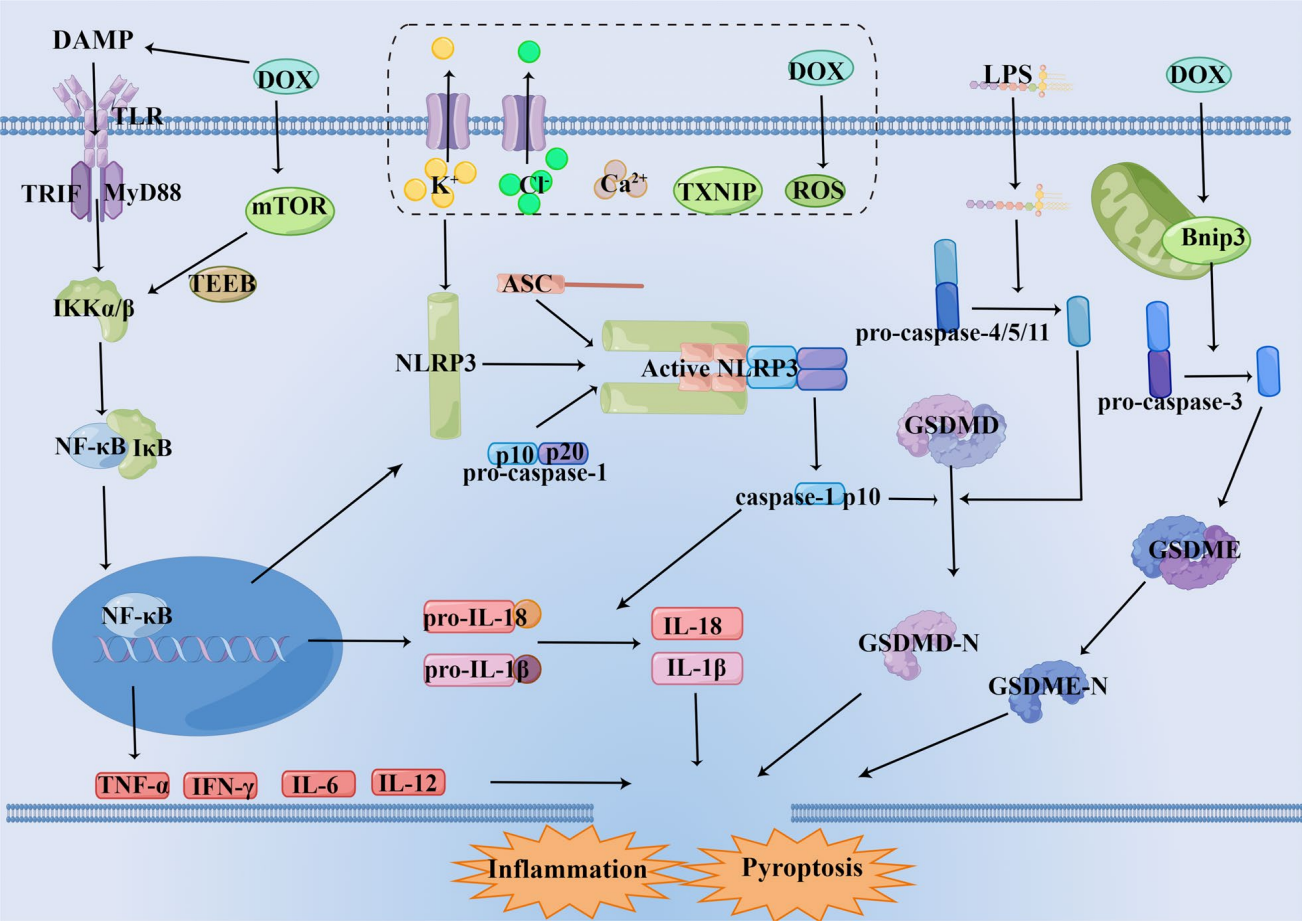
Schematic representation of the mechanism of pyroptosis and inflammation in DOX-induced cardiomyopathy. After Toll-like receptors (TLR) activation, it can transduce signals through myeloid differentiation factor 88 (MyD88) or Toll/IL-1 receptor domain-containing adapter-inducing interferon-β (TRIF), activate nuclear factor (NF-κB) and induce transcriptional upregulation of inflammasome regulators nucleotide-binding domain-like receptor protein 3 (NLRP3), leading to more efficient inflammasome assembly and release of damage-associated molecular pattern (DAMPs) such as IL-1β, IL-18, TNF-α and IFN-γ, mediating the inflammatory response. DOX can activate NLRP3 to assemble with ASC and pro-caspase-1, and further activating Caspase-1. Caspase-1 has both the p20 and p10 protein domains and generates activity via self-processing into Caspase-1p10 form. Activated caspase-1 cleaves gasdermin D (GSDMD) and causes GSDMD cleavage to produce N and C terminal fragments. GSDMD N terminus binds with the plasma membrane to form transmembrane pores, causing cell swelling and rupture, releasing numerous inflammatory factors into the blood system and inducing cell pyroptosis. In addition, DOX can upregulate Bnip3 to activate caspase-3 and cleave gasdermin E(GSDME) to produce the N and C terminal fragments, inducing cell pyroptosis. (By Figdraw.)

Inflammatory response is the defensive response of living tissues with vascular systems to various stimuli, including septic and aseptic inflammation. Aseptic inflammation is mainly caused by non-pathogenic stimuli such as physical and chemical conditions. In recent years, many studies have found that DIC is associated with aseptic inflammation [162]. DOX upregulates the levels of a variety of inflammatory factors, including interleukin-1 (IL-1) and tumor necrosis factor-α(TNF-α) in the heart, activating inflammatory and immune responses and leading to cardiomyocyte damage [163]. Zhang et al. found that there were two types of macrophages acting together to coordinate the inflammatory response in DIC, namely repairing macrophages and pro-inflammatory macrophages. A large number of lipid peroxides induced by DOX act as ligands to activate class A1 Scavenger receptors (SR-A1), activate transcription factor c-Myc by transforming growth bringing about the accumulation of Fe^2+^ within the mitochondria factor-activated kinase 1 and P38-related pathways, and further mediates the activation of SIRT1, influences the expression of macrophage self-renewal genes, promotes the proliferation of cardiac resident repair macrophages, and alleviates DIC [164]. However, this repair effect is insufficient to resist the inflammatory induced by DOX. The release of inflammatory factors is regulated by the following signaling pathways (Fig. 6).

### NLRP3/caspase-1/GSDMD signaling pathway

Pyroptosis is a cell death which is distinguished from apoptosis and is characterized by promoting inflammation and cell swelling or lysis [165], accompanied with massive release of by inflammatory factors and cellular contents [166]. Pyroptosis can be activated by the following three signaling pathways. One is the typical inflammasome pathway, which activates caspase-1 (caspase-1) and gasdermin D (GSDMD) by nucleotide-binding domain-like receptor protein 3 (NLRP3) [167]. The second is an atypical inflammasome pathway that occurs during pyroptosis during cell infection, resulting by the binding of caspase-4/5/11 through specific recognition of bacterial lipopolysaccharide [168]. The third is induced by activation of caspase-3 cleavage gasdermin E(GSDME) [169]. It has been found that DOX can induce pyroptosis through the above one and third paths, leading to myocardial damage.

NLRP3 is a significant regulatory factor of the innate immune system, whose activation requires the assembly of NLRP3 protein, apoptosis-associated speck-like protein containing a CARD (ASC), and caspase-1 into a mixture. The NLRP3 inflammasome can be activated by a variety of stimuli, including K^+^ excretion, Cl^−^ excretion, Ca^2+^ mobilization, mitochondrial dysfunction, lysosomal damage, the generation of ROS and so on [170]. The NLRP3 inflammasome can activate caspase-1 under the stimulation of endogenous and exogenous factors, on the one hand converting inactive IL-1β and IL-18 precursors into bioactive IL-1β and IL-18 and released outside the cell, leading in inflammatory and tissue damage. On the other hand, activated caspase-1 causes GSDMD lysis to produce N and C terminal fragments, and the N terminus of GSDMD binds to the plasma membrane, causing cell swelling and rupture, and a large number of inflammatory factors enter the blood system, leading to cell pyroptosis [171, 172]. GSDMD is a porogenic protein containing an N and a C terminal domain, which is one of the substrates of caspase-1 and plays a significant role in pyroptosis [173]. Caspase-1 has a conserved protease domain, which is divided into P20 and P10 subunits. The breakage of both subunits prompted activation of caspase-1. Caspase-1 can be self-processed into an active Caspase-1 p10 form, that binds to the C-terminal domain of GSDMD through hydrophobic interaction, cleaves GSDMD, exposes the GSDMD N terminus, and binds to the plasma membrane to form transmembrane pores, leading to cell swelling and rupture to mediate pyroptosis [174]. ASC does not participate in GSDMD cleavage, but can increase the amount of GSDMD, thereby enhancing GSDMD activation [175].

Due to the generation of ROS, reduced intracellular K^+^ levels and/or downregulation of Sirt1, DOX activates the NLRP3 inflammasome in cardiomyocytes and macrophages to activate caspase-1 as well as produce and secrete large amounts of pro-inflammatory cytokines, such as IL-1β, IL-18 and so on. IL-1β and IL-18 signaling triggers cardiomyocyte apoptosis through the IL-1 type I receptor (IL-1R), further promoting poor cardiac remodeling and inducing heart failure [176]. It was found that DOX treatment significantly upregulated the expression of NLRP3 and GSDMD, as well as the secretion of inflammatory cytokines in cardiomyocyte [177].

In the research of Wei et al., both H9c2 cells and animals treated with DOX showed upregulation of NLRP3 and caspase-1 p20, and elevated levels of caspase-1 and IL-1β and IL-18. Moreover, MCC950(a specific NLRP3 inhibitor) treatment reversed DOX-induced NLRP3 inflammasome activation and apoptosis in vitro and in vivo, proving that NLRP3 is involved in DIC [178]. Meng et al. experimentally demonstrated that DOX can promote NLRP3 expression by activating terminal differentiation-induced terminal differentiation-induced non-coding RNA (TINCR), increase the activity of NLRP3/caspase-1, and increase cardiomyocyte pyroptosis, thus leading to cardiac damage and dysfunction [179]. Thioredoxin interactive protein (TXNIP) overexpression has promoting effect on the activation of NLRP3. Honokiol can inhibit NLRP3 activation by inhibiting TXNIP expression [180]. Moreover, Nrf2 can also affect NLRP3 activation. It was found that Selenium and Pinocembrin could reduce the inflammatory response by enhancing Nrf2 expression and weakening the NLRP3 activation caused by DOX [181, 182]. Calycosin and Dihydromyricetin could inhibit inflammation and improve DIC by improving SIRT1, NLRP3 and related protein levels in cells and mouse hearts [183, 184]. Fraxetin, resveratrol, Nicotinamide mononucleotide and others exert anti-inflammatory effects to protect the heart by inhibiting NLRP3 activation and reducing the subsequent inflammatory factor secretion and release [185–188]. Zhang et al. found that Calycosin and MCC950 enhanced the viability of rat cardiomyocytes and attenuated DIC by inhibiting the NLRP3/caspase-1/GSDMD pathway [189, 190] (Additional file 5: Table S5).

Similarly, GSDME also produces a partial effect in cellular pyroptosis, as evidenced by the reduced doxorubicin-induced cellular pyroptosis upon knockdown of GSDME [191]. A variety of chemotherapeutic agents, including DOX, were found to trigger cell pyroptosis by activating caspase-3 and splitting GSDME [169]. As a classical anthracycline chemotherapeutic agent, DOX can promote JNK phosphorylation by increasing ROS accumulation, thereby inducing the activation of caspase-3, cleaving GSDME and triggering pyroptosis [192]. Zheng et al. have found that Bnip3 upregulated the expression of caspase-3 and lysis of the GSDME, thus alleviating doxorubicin-induced cell pyroptosis [191] (Additional file 5: Table S5).

### HMGB1/TLR4/MAPKs/NF-κB signaling pathway

Toll-like receptors (TLR), a class of transmembrane receptors, play a significant role in identifying various pathogen-associated molecular patterns and transducing signals into intracellular through their transmembrane regions [193]. Currently, 11 TLR species have been found in the human body, among which TLR2, TLR3, TLR4, and TLR5 play a role in DIC [194]. TLR4 is the first TLR found to be closely related to inflammation and is mainly expressed in immune cells [195]. TLR2, TLR4 and TLR5 are mainly expressed on the cell surface, whereas TLR3 is mainly expressed intracellularly. TLR can transduce signals dependent on or independent of the myeloid differentiation factor 88 (MyD88). TLR2 and TLR4 can bind to MyD88 to activate interferon regulator 3 (IRF3) and NF-κB, promoting the release of a series of damage-associated molecular pattern (DAMPs) [196, 197]. TLR2, TLR3 and TLR5 can also directly activate NF-κB, directly or indirectly promoting the secretion and release of inflammatory factors, being independent of MyD88 [198]. It was found that the ROS and RNS generated by DOX could upregulate TLR2, TLR4, and TLR5, and downregulate the expression of TLR3 [199, 200]. Animal experiments demonstrated that TLR4 deficiency and TLR5 deficiency attenuated doxorubicin-induced cardiac toxicity [201, 202], TLR2 deficiency suppressed the high expression of proinflammatory factors due to DOX [203].

It was found that HMGB1 takes part in the regulation of TLR4 expression. HMGB1 is a nuclear protein with pro-inflammatory effects, participating in the progress of autophagy, apoptosis, ferroptosis, inflammation etc. [204–207]. HMGB1, acted as a damage-associated molecular pattern (DAMP) protein, is secreted in response to ROS, RNS, Ca2 + and other stimuli, and is released extracellular by activated macrophages to mediate inflammatory response [208, 209]. HMGB1 released to extracellular cells can activate receptors such as TLR2 and TLR4, upregulate their expression, and further affect the release and secretion of inflammatory cytokines [209]. Study has found that HMGB1 levels were increased after DOX treatment, while silencing HMGB1 had detectable reduced TLR4 expression and reduced DIC due to DOX [210]. Thus, reducing HMGB1 can be a strategy to mitigate DIC. Zhang et al. found that rosuvastatin reduced the secretion of TNF-α and IFN-γ by reducing HMGB1 levels [211]. Du et al. found that miR-204 levels were decreased after DOX treatment, and miR-204 overexpression targeted HMGB1 to directly reduce its levels, thus exerting a cardioprotective effect [212]. Besides, myeloid differentiation protein 1(MD-1) appears to be related to the regulation of TLR4 expression and DOX-induced myocardial inflammation. Knockout of MD-1 can strengthen the activation of TLR4/MAPKs/ NF-κB pathway and aggravate DOX-induced myocardial inflammatory response [213].

The upregulation of TLR expression can significantly activate the TLR/MAPKs/NF-κB signaling pathway, and induce the expression of inflammatory cytokines such as interleukin 8 (IL-8) and TNF-α, leading to the injury and apoptosis of cardiomyocytes [67]. Mitogen-activated protein kinases (MAPKs) are mainly responsible for conducting signals from the cell surface to the nucleus, including ERK1/2, p38 and JNK. Nuclear factor (NF-κB), a protein complex, plays a significant role in regulating cellular inflammatory, involved in regulating the transcription of various pro-inflammatory cytokines [214]. NF-κB binds to IκB-α in normal physiological conditions and is not biologically active. When the body is endogenous or exogenous stimulated, it can interact with the IκB kinase (IKK) complex to promote IκB-α phosphorylation and accelerate the ubiquitination and degradation of IκB-α. The degradation of IκB-α can dissociate NF-κB from the NF-κB/IκB-α complex, increase the free NF-κB content, and transfer to the nucleus to generate activity [215]. It was found that Pristimerin and Nerolidol can exert anti-inflammatory effects and alleviate cardiac injury by inhibiting MAPKs/NF-κB signaling and subsequently inhibiting the effects of inflammatory cytokines [60, 216, 217].

Multiple compounds that exert anti-inflammatory effects by acting on TLR signaling were identified (Additional file 6: Table S6). LCZ696 (sacubitril/valsartan) attenuates DIC by inhibiting signaling from TLR2-MyD88 [203]. Enalapril and Crocin exert their cardioprotective effects by inhibiting the TLR-2/NF-κB pathway [218, 219]. Vanillic acid, and Ozone were found to reduce inflammatory cytokine release and reduce DIC by inhibiting the TLR-4/NF-κB pathway [220, 221]. It was found that Hemin alleviates inflammation by inhibiting the TLR-5/NF-κB/TNF-α pathway [199].

### mTOR/TFEB/NF-κB signaling pathway

Mechanistic target of rapamycin (mTOR), an atypical serine/threonine kinase, takes part in the regulation of various cellular functions, including mammals' growth and proliferation. The transcription factor EB (TFEB) with potential anti-inflammatory effects joins in regulating basic cellular processes [222]. NF-κB binds to IκB (NF-κB inhibitor) in the resting state, present in the cytoplasm, and is not bioactive. Upon stimulation by pro-inflammatory signals, IKKs are activated to activate NF-κB dimers and release and transport them to the nucleus, enhancing the expression of IL-8, TNF-α and other inflammatory cytokines [112]. The mTOR-mediated phosphorylation negatively regulates TFEB nuclear translocation and activity [223]. Reduced TFEB expression of TEEB can bring about an upregulation of IKK-α/βand NF-κB phosphorylation, which were reversed by TFEB overexpression. Wang et al. found that dihydrotanshinone could inhibit NF-κB by regulating the mTOR/TFEB pathway in cardiomyocytes, thereby inhibiting the expression of inflammatory cytokines and relieve DIC [224]. Moreover, curcuminy can also exert anti-inflammatory effects by inhibiting inflammatory factor release by activating the mTOR pathway [188].

## Conclusion

As a classical anthracycline anticancer drug, DOX has no doubt about its anticancer effect, while its clinical application is greatly limited by the dose-dependent cardiotoxicity—have made DOX a representative drug responsible for the study of anthracycline-induced cardiotoxicity. Currently, much progress has been made regarding the mechanism and clinical manifestations of cardiotoxicity caused by DOX. However, accurate prediction as well as effectively providing cardioprotection for patients vulnerable to DIC remains a mystery. In the past decades, there have been many researchers trying to find drugs that provide cardiac protection through the in vitro and in vivo experiments. In vitro, the investigators used a medium containing DOX to culture H9C2 cells for 24 h to construct a DOX cardiotoxicity model. In vivo, the experimental animals are often rats or mice. A model of DOX-induced acute cardiotoxicity was constructed by administering a disposable toxic dose of DOX, and the commonly used dose was a one-time intraperitoneal injection of 15–20 mg/kg DOX. Successful modeling was determined by measuring the presence of cardiac enzyme levels, echocardiography, HE staining of cardiac tissue, etc.

Many studies have been conducted on the molecular mechanisms of DIC, involving oxidative stress, inflammation, apoptosis, autophagy, mitochondrial damage, iron death, endoplasmic reticulum stress, Ca^2+^ overload and so on. DIC is a complex process resulting by the effects of multiple mechanisms. ROS generation is the result of redox metabolism after DOX enters the body, with ROS acting as a blasting fuse, and oxidative stress serving as the basis for other molecular mechanisms of DIC. On the one hand, overgeneration and accumulation of ROS directly damaged DNA and mitochondrial protein, causing mitochondrial dysfunction and tissue damage. On the other hand, ROS can also act as a signal to activate the body defense mechanisms to induce various ways of cell death, including apoptosis, autophagy, necrosis, pyroptosis, iron death, etc. Furthermore, oxidative stress and inflammatory responses are mutually causal and promote each other, exacerbating DIC. Inflammatory cytokines can participate in the generation of ROS. Simultaneously, ROS can induce NF-κB nuclear transcription factor activation and indirectly upregulate the level of inflammatory factors, making their overexpression, thus aggravating the inflammatory response of the body.

In conclusion, DOX induces overgeneration of ROS and RNS and leads to oxidative stress by activating Nrf2/Keap1/ARE, SIRT1/p66Shc, Sirt1/PPAR/PGC-1α pathway as well as interfering with NOS, NOX and Fe^2+^ signaling. DOX increases the secretion and release of inflammatory cytokines by acting on NLRP3/caspase-1/GSDMD, HMGB1/TLR4/MAPKs/NF-κB, mTOR/TFEB/NF-κB pathway, and further cause cell and tissue damage. It is necessary to further search for the molecular mechanism of DIC. Clarify the molecular mechanism of DIC can provide effective basis and ideas for the prevention and treatment of DIC and bring good news to cancer survivors.

## Future perspective

Numerous studies have shown that oxidative stress and inflammation are closely related to DIC. Studies have shown that the administration of antioxidant and anti-inflammatory therapy can reduce the level of myocardial damage markers and reduce DIC. However, antioxidants are hardly successful in the clinical prevention and treatment of DIC [225]. Although current antioxidant and anti-inflammatory treatments have not shown better therapeutic effects in DIC, inflammation and oxidative stress remain the focus of intensive research.

In recent years, researchers have paid more and more attention to the role of inflammatory response in cardiovascular diseases, which is recognized as a chronic process with inflammatory characteristics [226]. Immunomodulatory and specific anti-inflammatory therapies have been demonstrated in clinical trials to treat cardiovascular disease and mitigate cardiovascular disease mortality [227]. The role of inflammation in DIC is gradually discovered, and the research on DIC is no longer focused on cardiomyocytes, but expanded to investigate cardiac resident macrophages, neutrophils, B cells, T cells, endothelial cells and even systemic inflammation [228]. In the existing studies, anti-inflammatory therapy targeting TNF-α, pro-inflammatory cytokines has failed to show better efficacy, and finding more effective inflammatory targets is imminent [229]. Targeting immune cells (including heart resident macrophages, B cells, T cells, etc.) and regulating the body's immune function can be used as a new research idea. The study of immune system related to cardiac inflammation, especially cardiac resident macrophages may be the main research direction of DIC in the future [228, 230, 231]. In addition, various treatment that induce cardiomyocyte proliferation and cardiac regeneration, such as the transplantation of human embryonic stem cell-derived cardiac myocytes (hESC-CMs) and human induced pluripotent stem cell-derived cardiac myocytes (hiPSC-CMs), coding and non-coding gene inducers, small molecules inducer and so on, may also be the main research direction of DIC in the future [232].

## Supplementary Information

**Additional file 1. Table S1:** Some drugs that exert cardioprotective effects by acting on the Nrf2 / keap1 / ARE signaling pathway.

**Additional file 2. Table S2:** Some drugs that exert cardioprotective effects by acting on the NOX signaling.

**Additional file 3. Table S3:** Some drugs that exert cardioprotective effects by acting on the NOS signaling.

**Additional file 4. Table S4:** Some drugs that exert cardioprotective effects by acting on the PPAR/PGC-1α signaling pathway.

**Additional file 5. Table S5:** Some drugs that exert cardioprotective effects by acting on the NLRP3 signaling.

**Additional file 6. Table S6:** Some drugs that exert cardioprotective effects by acting on the TLR signaling.

**Additional file 7. Table S7:** Acting on some drug targets mitigates the oxidative stress and inflammation in DOX-induced cardiotoxicity.

## Data Availability

Not applicable.

## Abbreviations

^·^OH: Hydroxyl radical
ABCB8: ATP-binding cassette transporter protein B8
ACEI: Angiotensin converting enzyme inhibitor
Acot1: Acyl-coenzyme A thioesterase 1
ARB: Angiotensin receptor blocker
ARE: Antioxidant response element
ASC: Apoptosis-associated speck-like protein containing a CARD
BB: β-Blockers
BH4: Tetrahydrobiopterin
BNP: Type B brain natridium peptide
CK-MB: Creatine kinase isoenzyme
cTnI: Cardiac troponin I
DEX: Dexrazoxane
DIC: Doxorubicin-induced cardiomyopathy
DOX: Doxorubicin
Drp1: Dynamin-related protein-1
eNOS: Endothelial nitric oxide synthase
FAD: Flavin adenine dinucleotide
Fer-1: Ferrostatin-1
FMN: Flavin mononucleotide
FtMt: Mitochondrial ferritin
GPX 4: Glutathione peroxidase 4
GSDMD: Gasdermin D
GSDME: Gasdermin E
H_2_O_2_: Hydrogen peroxide
HO-1: Heme oxygenase-1
IFN-γ: Interferon-gamma
IKK: IκB kinase
IL: Interleukin
iNOS: Inducible nitric oxide synthas
IRF3: Interferon regulator
IRP: Iron regulins protein
JNK: C-Jun N-terminal kinase
Keap1: Kelch-like ECH associated protein 1
MAPK: Mitogen-activated protein kinases
MD-1: Myeloid differentiation protein 1
MRA: Mineralocorticoid receptor antagonist
mTOR: Mammalian target of rapamycin
MyD88: Myeloid differentiation factor 88
NAD (P) H: Nicotinamide adenine dinucleotide phosphate
NLRP3: Nucleotide-binding domain-like receptor protein 3
nNOS: Neuronal nitric oxide synthase
NOX: NAD (P) H oxidase
NQO-1: NAD (P) H quinone oxidoreductase-1
NRF-1: Nuclear respiratory factor 1
Nrf2: Nuclear Factor Erythroid 2-related Factor 2
O_2_^·−^: Superoxide
ONOO^−^: Peroxynitrite anion
P66Shc: The 66-kDa Src homology 2 domain-containing protein
PGC-1α: Peroxisome proliferator-activated receptor-gamma co-activator-1alpha
PKC: Protein kinase C
PRMT4: Protein arginine methyltransferase 4
RNS: Reactive nitrogen species
ROS: Reactive oxygen species
sGC: Soluble guanylate cyclase
sMaf: Small Maf proteins
SOD: Superoxide dismutase
SR-A1: Class A1 Scavenger receptors
TFAM: Mitochondrial transcription factor A
TFEB: Transcription factor EB
TfR: Transferrin receptor
TLR: Toll-like receptor
TRIM21: Tripartite motif containing-21
TXNIP: Thioredoxin interactive protein
UCP2: Uncoupling protein 2
