# Supplementary material for: Role of oxidative stress and inflammation-related signaling pathways in doxorubicin-induced cardiomyopathy
Source: Cell Commun Signal. 2023 Mar 14;21:61. doi: 10.1186/s12964-023-01077-5 (PMC10012797; doi:10.1186/s12964-023-01077-5)
Supplement: Supplementary file 2 — Additional file 1. Table S1: Some drugs that exert cardioprotective effects by acting on the Nrf2 / keap1 / ARE signaling pathway. [file 12964_2023_1077_MOESM2_ESM.docx]

| Compound | Model | Usage and dosage of drugs | Usage and dosage of DOX | Mechanism | Reference |
| --- | --- | --- | --- | --- | --- |
| dioscin | H9c2 cell  mice  rats | 0-1000ng/mL,for 24h  80,40,20mg/kg,PO,for 7 days  60,30,15mg/kg,PO,for 7 days | 5 μM, for 24 h  15mg/kg,IP,once  15mg/kg,IP,once | miR-140-5p(-)  Sirt2/Nrf2(+) | [41] |
| α-Linolenic acid | rats | 500 μg/kg,PO, for 17 days | 2.5 mg/kg, IP, for 7 times in 14 days | Keap1/Nrf2(+) | [48] |
| sulforaphane | rats | 0.5 mg/kg,IP | 2.5 mg/kg, IP, for 6 times in 12 days | Nrf2(+) | [49] |
| asiatic Acid | rats | 20 mg/kg, PO, for 7 days | 65.75 mg/kg, iv, once | Nrf2(+) | [50] |
| curdione | H9c2 cell | 100µM,for 24h | 1.25 µM,for 24h | Nrf2/HO-1(+) | [34] |
| shengmai injection | rats  H9c2 cell | 2.7,5.4,10.8mL/kg,IP,for 7days  2 mL,for 24h | 15 mg/kg,IP,once  1 M DOX,for 24h | Nrf2/HO-1(+) | [61] |
| ganoderma lucidum polysaccharides | male rats  H9c2 cell | 50,100 mg/kg,PO  100、50μg/ml | 2 mg/kg,IP, for 3 times in 6 days  1 μM,for 6h | Nrf2(+) | [53] |
| p-coumaric acid | H9c2 cell | 380.63 μM, for 24h | 1.5 μM, for 24h | Nrf2/ARE(+) | [54] |
| resolvin D1 | rats | 2.5 μg/kg,IP | 20 mg/kg,IP,once | Nrf2/HO-1(+),  NOX2/NOX4(-) | [55] |
| punicalagin | H9c2 cell | 50,100,200μM,for 24h | 5 μM,for 24h | Nrf2/HO-1(+) | [57] |
| fisetin | rats | 20,40 mg/kg/day,IP | 2.5 mg/kg, IP, for 6 times in 6 weeks | Sirt1/Nrf2(+) | [39] |
| mokko lactone | rats | 30 mg/kg,IP | 15mg/kg,IP,once | Nrf2/HO-1(+) | [59] |
| baicalein | mice | 25,50mg/kg,PO,for 15 days | 5 mg/kg, IP, for 3 times in 15 days | Nrf2/HO-1(+) | [62] |
| beta-LAPachone | mice | 2.5,5mg /kg ,PO | 15mg /kg , IP,once | Nrf2/HO-1(+) | [63] |
| nerolidol | rats | 50mg/kg/d,PO,for 5 weeks | 2.5mg/kg/w,IP,for 5 times in 5 weeks | Nrf2/HO-1(+)  MAPK/NF-κB(-)  TNF-α、IL-6、IL-1β(-) | [60] |
| 3,3'-diindolylmethane | mice | 10,25,40,80 mg/kg b.w. for 28 days | 5 mg/kg, IP, for 4 times in 8 days | Nrf2/ARE(+) | [64] |
| tanshinone IIA | mice | 15 mg/kg,IP | 18 mg/kg,IP,once | Nrf2/ARE(+) | [65] |
| cardamonin | mice | 20,40,80mg/kg/day,IP | 5mg/kg/week,IP,for 4 weeks | Nrf2/ARE(+) | [67] |
| danshensu | mice | 50,100mg/kg/day, IP | 15 mg/kg,IP,once | Keap1/Nrf2/NQO1(+) | [68] |
| limonin | rats | 5/10 mg/kg/d,IP | 10 mg/kg,IP,once | Sirt2/Nrf2(+) | [35] |
| β-Hydroxybutyrate | mice  H9c2 cell | 10 mmol/kg/d,IP, for 5 times a day  10 mM,for 24h | 5mg/kg/d,IP,for 4 times in 4 days  1μM,for 24h | Nrf2(+)  SOD(+) | [69] |

**Table S1: Some drugs that exert cardioprotective effects by acting on the Nrf2 / keap1 / ARE signaling pathway.** Sirt: Silent information regulator, Nrf2: Nuclear factor E2-related factor 2, Keap1: kelch-like ECH associated protein 1, HO-1: heme oxygenase-1, ARE: antioxidant response element, SOD: superoxide dismutase, NQO-1: NAD(P)H quinone oxidoreductase-1, NOX: NAD(P)H oxidase, MAPK: mitogen-activated protein kinases, NF-κB: nuclear factor-kappaB, TNF-α: tumor necrosis

factor-α, IL:interleukin, IP: intraperitoneal injection, iv: intravenous injection.
