# Supplementary material for: Role of oxidative stress and inflammation-related signaling pathways in doxorubicin-induced cardiomyopathy
Source: Cell Commun Signal. 2023 Mar 14;21:61. doi: 10.1186/s12964-023-01077-5 (PMC10012797; doi:10.1186/s12964-023-01077-5)
Supplement: Supplementary file 3 — Additional file 2. Table S2: Some drugs that exert cardioprotective effects by acting on the NOX signaling. [file 12964_2023_1077_MOESM3_ESM.docx]

**Table S2:** **Some drugs that exert cardioprotective effects by acting on the NOX signaling.** NOX:NAD(P)H oxidase, NF-κB: nuclear factor-kappaB, ROS: reactive oxygen species, AngII: Angiotensin II, MAPK: mitogen-activated protein kinases, IP: intraperitoneal injection.

| Compound | Model | Usage and dosage of drugs | Usage and dosage of DOX | Mechanism | Reference |
| --- | --- | --- | --- | --- | --- |
| neferine | H9c2 cell | 1-20 µM,for 24 h | 0.1- 50 µM, for 24h. | NOX2(-)  P22phox,p67phox(-)  AngII(-)  NF-κB(-) | [21] |
| valsartan | rats | 20mg/kg/week,IP,for 6 weeks | 2.5mg/kg/week,IP,for 6 weeks | NOX2/NOX4(-) | [85] |
|  | H9c2 cell | 5 µM, for 1h | 1 µM, for 24 h | AngII/NOX/ROS/MAPK(+) | [87] |
| necrostatin-1 | rats | 1.65 mg/kg/d,IP, for 7 days | 10mg/kg,IP,once | NOX2(-) | [90] |
| setanaxib | mice | 60 mg/kg/d,PO | 5 mg/kg/w,IP,for 4 times in 3 weeks | NOX4(-)  ROS(-) | [91]. |
| astragaloside IV | mice | 40mg/kg/week,IP,for 4 weeks | 8mg/kg/week,IP,for 4 weeks | NOX2(-)  NOX4(-) | [88] |
| acacia hydaspica | rats | 200,400mg/kg/day,PO,for 6 weeks | 3 mg/kg/week,PO,for 6 weeks | oxidative stress(-)  P22phox,p47phox(-)  ROS(-) | [89] |
| irisin | mice | 12nmol/kg/day,IH,for 14 days | 15 mg/kg,IP,once | p67phox(-)  NOX(-) | [94] |
