# Supplementary material for: Role of oxidative stress and inflammation-related signaling pathways in doxorubicin-induced cardiomyopathy
Source: Cell Commun Signal. 2023 Mar 14;21:61. doi: 10.1186/s12964-023-01077-5 (PMC10012797; doi:10.1186/s12964-023-01077-5)
Supplement: Supplementary file 4 — Additional file 3. Table S3: Some drugs that exert cardioprotective effects by acting on the NOS signaling. [file 12964_2023_1077_MOESM4_ESM.docx]

**Table S3:** **Some drugs that exert cardioprotective effects by acting on the NOS signaling.** iNOS: inducible nitric oxide synthas, TNF-α: tumor necrosis factor-α, GPX: glutathione peroxidase, SOD: superoxide dismutase, IL:interleukin, GSH: glutathione, NF-κB: nuclear factor-kappaB, eNOS: endothelial nitric oxide synthase, ROS: reactive oxygen species. IP: intraperitoneal injection.

| Compound | Model | Usage and dosage of drugs | Usage and dosage of DOX | Mechanism | Reference |
| --- | --- | --- | --- | --- | --- |
| phenylalanine-butyramide | mice | 30mg/kg/day,PO,for 21 days | 1 mg/kg/day,IP,for7 days | iNOS(-) | [99] |
| eicosapentaenoic acid,vitamin E. | rats | 300mg/kg/d;PO,for 14 days.  200mg/kg/d;PO,for 14 days. | 15 mg/kg,IP,once | iNOS(-),  TNF-α(-),  GPX、SOD(+), | [103] |
| fisetin | rats | 10、20、40 mg/kg,for 7 days | 15 mg/kg,IP,once | iNOS(-)  IL-1、TNF-α(-)  SOD、 GSH(+) | [105] |
| curcumin | rats | 100mg/kg,PO,for 15 days | 20 mg/kg,IP,once | iNOS(-)  NF- κB/TNF α(-) | [104] |
| crocin | rats | 100mg/kg/day,PO,for 3 weeks | 3.5mg/kg,Twice a week,IP,for 3 weeks | NF- κB/TNF α(-)  iNOS(-) | [106] |
| nebivolol | rats | 4mg/kg,IP,for 7 times in 2 weeks | 2.5 mg /kg,IP,for 7 times in 2 weeks | eNOS uncoupling(-)  iNOS(-)  TNF-α(-) | [107] |
| vitamin C | Cardiomyocytes from rats | 25µM, for 1 h | 10 µM, for 24 h | eNOS uncoupling(-)  TNF-α、IL-1β、IL-6(-) | [110] |
| vitamin C | rats | 50mg/kg/day,PO | 2.5mg/kg,IP,for 6 times in 3 weeks | TNF-α、IL-1β(-)  eNOS uncoupling(-) | [112] |
| a special amino-acid formula tailored | mice | 1500mg/kg/day,po | 20 mg/kg,IP,once | eNOS uncoupling(-) | [113] |
| fenofibrate | mice | 20mg/kg/d,for 4 weeks | 4mg/kg/week,for 5 times in 5 weeks | Akt/eNOS(-) | [115] |
| ursolic acid | mice | 80mg/kg/d,IH,for 7 days | 15 mg/kg,IP,once | ROS(-)  NOX4(-)  eNOS uncoupling(-) | [116] |
| folic acid | mice | 10mg/day,PO,for 7 days | 20mg/kg,IP,once | SOD(-)  eNOS uncoupling(-) | [117] |
| fluvastatin | rats | 6 mg/kg/d,PO,for 7 days | 7.5 mg/kg,IP, for 3 times in 7 days | eNOS uncoupling(-)  iNOS(-)  NF-κB(-) | [108] |
