# Supplementary material for: Role of oxidative stress and inflammation-related signaling pathways in doxorubicin-induced cardiomyopathy
Source: Cell Commun Signal. 2023 Mar 14;21:61. doi: 10.1186/s12964-023-01077-5 (PMC10012797; doi:10.1186/s12964-023-01077-5)
Supplement: Supplementary file 5 — Additional file 4. Table S4: Some drugs that exert cardioprotective effects by acting on the PPAR/PGC-1α signaling pathway. [file 12964_2023_1077_MOESM5_ESM.docx]

**Table S4: Some drugs that exert cardioprotective effects by acting on the PPAR/PGC-1α signaling pathway.** PPAR: Peroxisome proliferator-activated receptors, PGC-1α: Peroxisome proliferator-activated receptor-gamma co-activator-1alpha, NF-κB: nuclear factor-kappaB, GPX: glutathione peroxidase, SOD: superoxide dismutase, Sirt: Silent information regulator, Nrf2: Nuclear factor E2-related factor 2, TNF-α: tumor necrosis factor-α, IL:interleukin, UCP-2: uncoupling protein 2, iNOS: inducible nitric oxide synthas, IP: intraperitoneal injection.

| Compound | Model | Usage and dosage of drugs | Usage and dosage of DOX | Mechanism | Reference |
| --- | --- | --- | --- | --- | --- |
| astragalus polysaccharide | rats | 0.5mg/kg,IV | 2.5mg/kg/week,IP,for 6 times in 6 weeks | PPAR-γ(-)  NF-κB(-)  SOD、GPX(+) | [120] |
| troxerutin | rats | 150mg /kg/d,PO,for 4 weeks | 20 mg/kg,IP,once | SIRT1/ PGC-1α(+)  Nrf2(+) | [121]. |
| glycyrrhiza glabra | H9c2 cell | 20—200µg/ml | 1-5µM,for 24h | PPAR-α(+)  PPAR-γ(+) | [124] |
| piperine | mice | 50mg/kg/d,PO,for 3 weeks | 15mg/kg,IP,once | PPAR-γ(+) | [126] |
| astragali radix | mice  H9c2 cell | AR water extract as freedrinking water  400–1600μg/mL,for 6 h | 3mg/kg,IP,for 8 times in 2 weeks  0.5μM,for 24 h | PPAR-α(+)  PPAR-γ(+) | [127] |
| catalpol | H9c2 cell | 0, 10, 20, 40 ,80 µM for 24 h | 0,0.1, 1 ,10 µM for 12 and 24 h | TNF-α, IL-1β, IL-6(-)  PPAR-γ(+) | [128] |
| ferruginol | mice | 20 mg/kg,PO,for 4 weeks | 5 mg/kg/w,IP,for 4 times in 4 weeks | SIRT1/PGC-1α(+) | [132] |
| dichloroacetate | mice | 200 mg/kg/d,IP | 20 mg/kg,IP,once | SIRT3/PGC-1α(+) | [133] |
| pterostilbene | mice  H9c2 cell | 10mg/kg/d,IP,for 7 days  10 μM | 10mg/kg,IP,for 2 times in 4 days  1 μM ,for 24h | AMPK/SIRT1/PGC-1α(+) | [134] |
| dexmedetomidine | mice | 50 μg/kg,IP | 20mg/kg,IP,once | PGC-1α(+)  UCP2(+) | [138] |
| fidarestat | THP-1 human monocyte  mice | 30 μM,for 24h  25mg/kg/d,PO,for 3 weeks | 0.2,0.5,1μM,for 24h  4mg/kg/w,for 3 times in 3 weeks | PGC-1α(+)  IL-6、IL-1β(-)  iNOS(-) | [136] |
