# Supplementary material for: Role of oxidative stress and inflammation-related signaling pathways in doxorubicin-induced cardiomyopathy
Source: Cell Commun Signal. 2023 Mar 14;21:61. doi: 10.1186/s12964-023-01077-5 (PMC10012797; doi:10.1186/s12964-023-01077-5)
Supplement: Supplementary file 6 — Additional file 5. Table S5: Some drugs that exert cardioprotective effects by acting on the NLRP3 signaling. [file 12964_2023_1077_MOESM6_ESM.docx]

**Table S5: Some drugs that exert cardioprotective effects by acting on the NLRP3 signaling.** Sirt: Silent information regulator, NLRP3: nucleotide-binding domain-like receptor protein 3, GSDMD: gasdermin D, Nrf2: Nuclear factor E2-related factor 2, SOD: superoxide dismutase, IL:interleukin, GSH: glutathione, TXNIP: Thioredoxin interactive protein, mTOR: Mammalian target of rapamycin, IP: intraperitoneal injection.

| Compound | Model | Usage and dosage of drugs | Usage and dosage of DOX | Mechanism | Reference |
| --- | --- | --- | --- | --- | --- |
| calycosin | H9c2 cell  H9c2 cell | 50, 100, and 200 μM,for 24h  5-160μg/mL | 5 μM, for 24 h  0.5-20μM | Sirt1/NLRP3/caspase1/ GSDMD(-) | [183, 189] |
| dihydromyricetin | rats | 100,200 mg/kg/day | 15mg/kg,IP,once | SIRT1/NLRP3(-) | [184] |
| selenium | mice | 0.2mg/kg,IP | 15 mg/kg,IP,once | Nrf2(+)  NLRP3(-) | [181] |
| pinocembrin | mice  H9c2 cell | 5mg/kg,IP,once every 2 days  1μM,for 48h | 20mg/kg,IP,once  1μM,for 48h | Sirt3/Nrf2(+)  NLRP3(-) | [182] |
| fraxetin | rats | 40,80 mg/kg/d,PO,for 2 weeks | 4mg/kg,IP,for 4 times in 2 weeks | NLRP3(-) | [186] |
| resveratrol | mice | 320 mg/kg/d,PO | 4mg/kg/w,IP,for 3 times in 3 weeks | NLRP3(-) | [185] |
| nicotinamide mononucleotide | rats | 500mg/kg,IP,once every 3 days | 2.5 mg/kg/w,IP, for 6 times in 6 weeks | NLRP3/Caspase-1(-)  GSH,SOD(+) | [187] |
| MCC950 | mice  H9c2 cell | 10mg/kg,IP,once every 2 days, for 4 weeks  7.5 nM,for 24h | 5 mg/kg/w,IP,for 4 weeks  1µM,for 24h | NLRP3/caspase-1/ GSDMD(-) | [190] |
| honokiol | H9c2 cell | 2.5 or 5.0 µM ,for 24 h. | 0.1 µM,for 48 h | TXNIP/ NLRP3(-) | [180] |
| curcumin | rats  H9c2 cell | 100,200,400mg/kg/d,PO  10 µmol/L,for 24h | 3mg/kg,IP,for 8 times in 16 days  20 µmol/L,for 24h | NLRP3/Caspase-1/IL-18(-)  PI3K/Akt/mTOR(+) | [188] |
