# Supplementary material for: Role of oxidative stress and inflammation-related signaling pathways in doxorubicin-induced cardiomyopathy
Source: Cell Commun Signal. 2023 Mar 14;21:61. doi: 10.1186/s12964-023-01077-5 (PMC10012797; doi:10.1186/s12964-023-01077-5)
Supplement: Supplementary file 7 — Additional file 6. Table S6: Some drugs that exert cardioprotective effects by acting on the TLR signaling. [file 12964_2023_1077_MOESM7_ESM.docx]

**Table S6:** **Some drugs that exert cardioprotective effects by acting on the TLR signaling.** TLR: Toll-like receptors, MyD88: myeloid differentiation factor 88, Nrf2: Nuclear factor E2-related factor 2, HO-1: heme oxygenase-1, ARE: antioxidant response element, MAPK: mitogen-activated protein kinases, NF-κB: nuclear factor-kappaB, HMGB1: high mobility group box 1, IP: intraperitoneal injection.

| Compound | Model | Usage and dosage of drugs | Usage and dosage of DOX | Mechanism | Reference |
| --- | --- | --- | --- | --- | --- |
| LCZ696 | mice | 60 mg/kg/d,PO | 5mg/kg/w,IP,for 3 times in 3 weeks | TLR2-MyD88(-) | [203] |
| cardamonin | mice | 20,40,80mg/kg,PO | 5mg/kg/w,IP,for 4 times in 4 weeks | Nrf2/ARE(+)  NF-kB(-) | [67] |
| pristimerin | rats | 0.5,1mg/kg/d,IP, for 1 week | 2.5mg/kg,IP,for 6 times in 2 weeks | Nrf2/ARE(+)  MAPK/NF-kB(-) | [216] |
| nerolidol | rats | 50 mg/kg,PO,for 5 days | 12.5 mg/kg,IP,once | Nrf2/Keap1/HO-1(+)  MAPK/NF-kB(-) | [217] |
| enalapril | rabbit | 5mg/kg,PO,for 8 weeks | 1mg/kg,IP,for 16 times in 8 weeks | TLR2/NF-kB(-) | [219] |
| vanillic acid | rats | 10,20,40mg /kg,PO,for 2 weeks | 2.5 mg/kg,IP,for 6 times in 2 weeks | TLR4(-) | [220] |
| ozone | H9c2 cell | 50 µg/mL ,for 24 h | 0.5, 1 , 1.5 , 2 µM,for 24 h | TLR4/NF-kB(-) | [221] |
| rosuvastatin | rats | 1mg/kg/d,PO,for 14 days | 1mg/kg/d,IP,for 14 days | HMGB1/TLR4/NF-κB(-) | [211] |
