# Supplementary material for: Role of oxidative stress and inflammation-related signaling pathways in doxorubicin-induced cardiomyopathy
Source: Cell Commun Signal. 2023 Mar 14;21:61. doi: 10.1186/s12964-023-01077-5 (PMC10012797; doi:10.1186/s12964-023-01077-5)
Supplement: Supplementary file 8 — Additional file 7. Table S7: Acting on some drug targets mitigates the oxidative stress and inflammation in DOX-induced cardiotoxicity. [file 12964_2023_1077_MOESM8_ESM.docx]

**Table S7:** **Acting on some drug targets mitigates the oxidative stress and inflammation in DOX-induced cardiotoxicity.** Nrf2: Nuclear factor E2-related factor 2, HO-1: heme oxygenase-1, Sirt: Silent information regulator, p66Shc: The 66-kDa Src homology 2 domain-containing protein, NOX: NAD(P)H oxidase, eNOS: endothelial nitric oxide synthase, PPAR: Peroxisome proliferator-activated receptors, PGC-1α: Peroxisome proliferator-activated receptor-gamma co-activator-1alpha, Drp1: Dynamin-related protein-1, GPX 4: glutathione peroxidase 4 , HMGB1: high mobility group box 1, TLR: Toll-like receptors, TNF-α: tumor necrosis factor-α, IL:interleukin,  IFN-γ: Interferon-gamma.

| Targets | Model | usage and dosage of DOX | mechanism | reference |
| --- | --- | --- | --- | --- |
| Orosomucoid 1(+) | rats  H9c2 cell | 20 mg/kg,IP,once  10  μM,for 24h | Nrf2/HO-1(+) | [56] |
| miR-200a | mice | 15mg/kg,IP,once | Nrf2(+) | [66] |
| miR-34a-5p | rats | 2 mg/kg,iv, twice a week for 4, or 8 weeks | Sirt1/p66shc(+) | [80] |
| miR-124 | mice | 3 mg/kg/w,IP, for 3 weeks | Sirt1/p66shc(+) | [81] |
| Rac1 | mice | 20 mg/kg, IP,once | Rac1(-)  NOX(-) | [93] |
| soluble Guanylate Cyclase(sGP) | mice | 20 mg/kg ,IP,once | sGP(+)  Stabilize eNOS | [109] |
| miR-128-3p(-) | mice | 15 mg/kg,IP,once | PPAR-γ(+) | [125] |
| miR-22 (-) | mice | 5 mg/kg/w,for 5 times in 5 weeks | sirt1/PGC-1α(+) | [135] |
| miR-130a(-) | H9c2 cell | 5µM,for 24h | PPARγ(+) | [140] |
| miR-23a(-) | Neonatal rats  ventricular myocytes(NRVMs) | 1, 3, or 5 μM for 24 h | PGC-1α/Drp1(+) | [141] |
| PRMT4(+) | mice | 15 mg/kg,IP,once | Nrf2/GPX4(+) | [149] |
| TRIM21(-) | mice | 20mg/kg,IP,once | Nrf2(+)  Ferroptosis(-) | [43] |
| ABCB8(+) | H9c2 cell | 10 μM,for 24 h. | Ferroptosis(-) | [160] |
| HMGB1(-) | H9c2 cell | 2 μM | HMGB1/TLR4/TNF-α/IFN-γ(-) | [210] |
| miR-204(+) | mice  H9c2 cell | 15mg/kg,IP,once  1μmol/L,24h | HMGB1(-)  TNF-α、IL-1、IL-6(-) | [212] |
| class A1 scavenger receptors(SR-A1) | mice | 2.5mg/kg,IP,for 6 times in 14 days | SR-A1/c-Myc (+) | [164] |
| MITOL/MARCH5 | mice  H9c2 cell | 20 mg/kg,IP,once  10 μM; for 24 h | GPX4(+) | [152] |
| miR-140-5p(-) | H9c2 cell | 0–10μM,for 24h | SIRT2/Nrf2(+) | [40] |
